# Quantitative analysis of DNA‐GATA1 binding alterations linked to hematopoietic disorders

**DOI:** 10.1111/febs.70483

**Published:** 2026-03-17

**Authors:** Boglarka Zambo, Bastien Morlet, Luc Negroni, Orsolya Mózner, Balázs Sarkadi, Gilles Travé, Gergo Gogl

**Affiliations:** ^1^ Équipe Labellisée Ligue contre le cancer Institut de Génétique et de Biologie Moléculaire et Cellulaire (IGBMC), INSERM U1258/CNRS UMR 7104/Université de Strasbourg Illkirch France; ^2^ Institut de Biologie Valrose (iBV) Inserm U1091/CNRS UMR7277/Université Côte d'Azur Nice France; ^3^ Institut de Génétique et de Biologie Moléculaire et Cellulaire (IGBMC) INSERM U1258/CNRS UMR 7104/Université de Strasbourg Illkirch France; ^4^ HUN‐REN Research Centre for Natural Sciences Institute of Molecular Life Sciences Budapest Hungary

**Keywords:** DNA‐binding, GATA1, hematopoiesis, mutations, quantitative interactomics

## Abstract

GATA1 is a crucial transcription factor involved in hematopoiesis and mutations in this gene are linked to severe hematological disorders, including anemia, thrombocytopenia, Down syndrome‐related transient abnormal myelopoiesis (DS‐TAM), and myeloid leukemia of Down syndrome (ML‐DS). Despite significant clinical interest in the molecular level characterization of GATA1 mutations, a comprehensive understanding of their impact on DNA binding is limited. Efforts to conduct detailed studies on full‐length recombinant GATA1 have faced significant technical challenges, while alternative approaches are limited by low throughput or qualitative nature. Here, we introduce a native holdup (nHU) assay designed to systematically quantify DNA‐protein interactions and is suitable for studying the impact of transcription factor mutations on DNA binding affinity. First, using the erythroid‐specific *ATP2B4* promoter as a model, we demonstrate that nHU can capture sequence‐specific interactions and detect even subtle differences in DNA binding affinities. Then, we quantitatively characterize the impact of pathological mutations on DNA binding affinities in the context of full‐length human GATA1. Our findings reveal that the GATA1s isoform, lacking the N‐terminal transactivation domain (N‐TAD), binds to DNA with increased affinity, while the R307C mutation reduces binding to the *ATP2B4* erythroid promoter. In harmony with these observations, GATA1s exhibits increased functional activity, while the R307C mutation results in decreased activity. This study demonstrates the power of the nHU assay for studying DNA interactions of transcription factor variants and providing insight into the molecular mechanism of related diseases.

## Graphical abstract

Native holdup allows the quantitative determination of affinities between full‐length transcription factors and DNA. Mutations in either the protein or the DNA can modulate binding strength, which can be precisely quantified using this approach. Applied to GATA1, it revealed mutations that alter DNA binding. This method provides a robust approach for elucidating mutation‐induced functional changes in transcription factors.

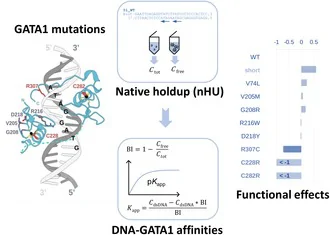

**Abbreviations**
4945: predicted GATA1 binding sequence at SNPs rs10751449/rs10736845
51: predicted GATA1 binding sequence at SNP rs10751451
AI: artificial intelligence
ATF2: activating transcription factor 2
ATP2B4: ATPase plasma membrane Ca^2+^ transporting 4
CF: C‐terminal zinc finger DNA‐binding domain
ChIP: chromatin immunoprecipitation
CNBP: cellular nucleic acid‐binding protein
dsDNA: double‐stranded DNA
DS‐TAM: Down syndrome‐related transient abnormal myelopoiesis
EMSA: electrophoretic mobility shift assay
FOG1: friend of GATA protein 1
HAP: haplotype
HEK: human embryonic kidney
HOXB4: homeobox protein Hox‐B4
ITC: isothermal titration calorimetry
LIN28B: Lin‐28 homolog B
LMO2: LIM domain only 2
ML‐DS: myeloid leukemia of Down syndrome
MS: mass spectrometry
NF: N‐terminal zinc finger DNA‐binding domain
nHU: native holdup
N‐TAD: N‐terminal transactivation domain
PMCA4b: plasma membrane calcium ATPase 4b
SCR: scrambled
SNP: single nucleotide polymorphism
SPR: Surface plasmon resonance
ssDNA: single‐stranded DNA
TAL1: T‐cell acute lymphocytic leukemia protein 1
TF: transcription factor
TMEM258: transmembrane protein 258
WB: western blot
WT: wild type
ZNF24: zinc finger protein 24

## Introduction

**Fig. 1:**
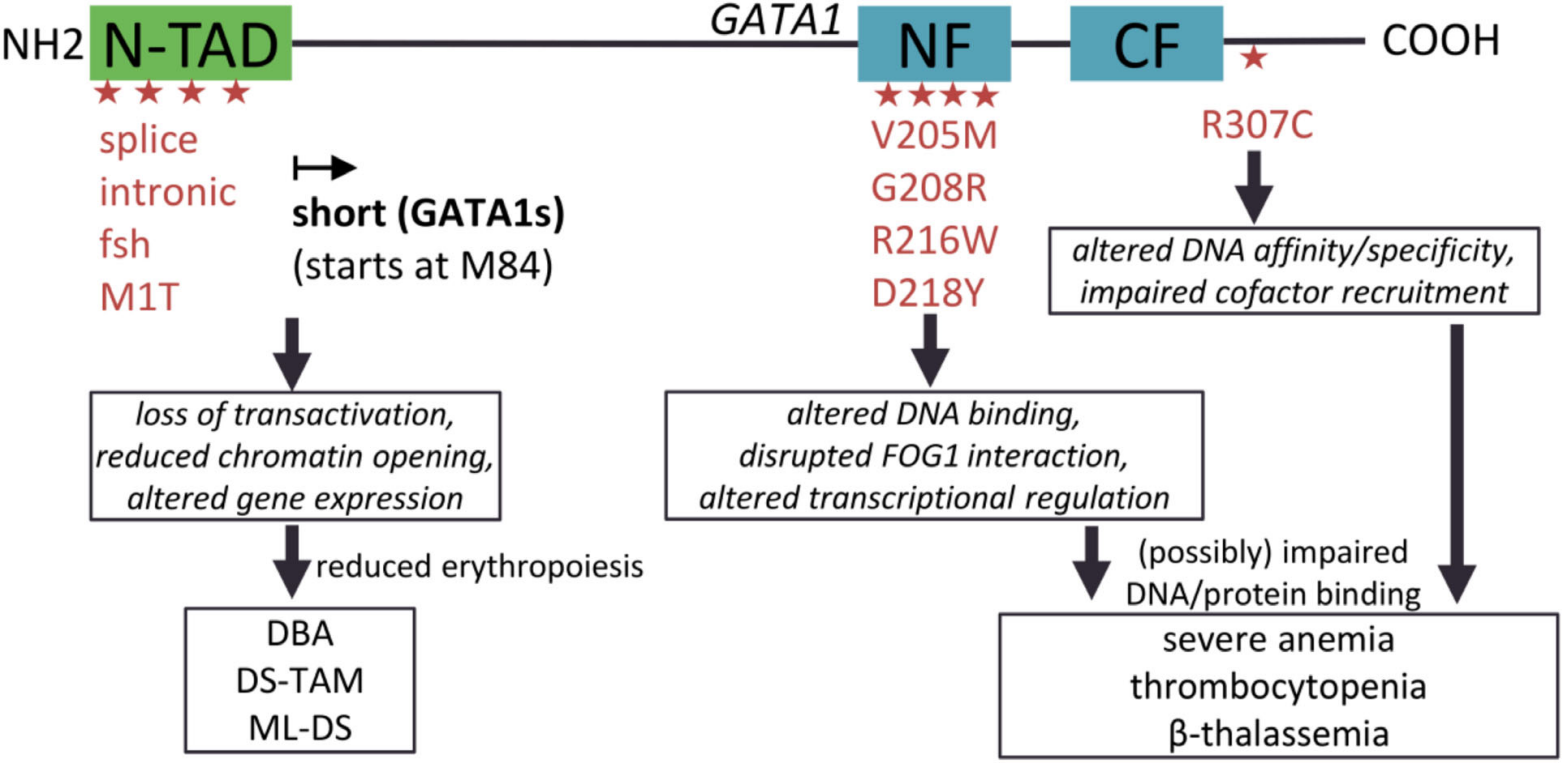
Implication of GATA1 mutations in diseases. Splicing defects, intronic, frameshift mutations in the first part of the gene or start codon mutation lead to the expression of the short isoform (GATA1s, starting from Met84) which is implicated in Diamond–Blackfan anemia (DBA), Down syndrome associated transient abnormal myelopoiesis (DS‐TAM) or myeloid leukemia of Down syndrome (ML‐DS). Mutations (labeled with red stars) in either the N‐terminal zinc finger (NF) or close to the C‐terminal zinc finger (ZF) are implicated in severe anemia, thrombocytopenia, and β‐thalassemia.

The erythroid transcription factor (TF) GATA1 is essential for the differentiation of myeloid progenitor cells to mature blood cells in the bone marrow [1, 2]. Complete loss of GATA1 in mice is embryonically lethal due to the absence of red blood cells and mutations in the gene disrupt normal erythropoiesis [3, 4, 5, 6]. GATA1 is composed of two C4‐type zinc fingers, known as the N‐terminal (NF) and C‐terminal (CF) fingers (Fig. 1) [7]. The CF binds to DNA with high affinity and specificity through (A/T)GATA(A/G) sequences, while the NF displays weaker DNA binding and instead plays a modulatory role by interacting with other proteins such as FOG1 or LMO2 [8, 9, 10].

One of the interesting potential target genes of GATA1 is *ATP2B4*, where an erythroid promoter is located just upstream of the second exon, driving the expression of the PMCA4b calcium pump specifically in erythroid lineages [11, 12]. In this erythroid promoter, a haplotype has been identified that causes lower expression of the protein in mature red blood cells, reduces mean corpuscular hemoglobin concentration in the blood, and provides a certain degree of protection against malaria [11, 13, 14, 15, 16]. We showed in our previous study that a significant increase in transcription can be detected once GATA1 is co‐transfected along with the reporter plasmid containing this erythroid promoter. Furthermore, by using site‐specific mutagenesis screens, we identified two SNP‐containing sites in this alternative promoter region referred to as 4945 and 51 (abbreviations for SNP identification codes rs10751449/rs10736845 and rs10751451), which are single and tandem GATA1 recognition sites altered by the haplotype [11]. In reporter assays where this erythroid promoter was inserted to drive the expression of a luciferase, the mutation of any or both of these SNPs was enough to recapitulate the reduced expression observed with the full haplotype sequence [17].

Over the past decades, many mutations have been identified in GATA1 from patients suffering from different cytopenia, such as severe anemia, thrombocytopenia, and/or β‐thalassemia (Fig. 1). These mutations are most commonly located in the NF (V205M, G208R/S, R216Q/W, D218G/N/Y) [18, 19, 20, 21, 22, 23, 24, 25]. Other mutations with similar clinical manifestations are located just downstream of the CF (insertion of PPFWQ at position 290 or R307C/H/L) [26, 27]. These mutations, positioned within or close to the zinc fingers, may affect DNA binding properties. However, no systematic study has yet investigated their impact on full‐length human GATA1 under physiological ionic strength.

In addition to point mutations, splicing and start codon defects—including intronic or splice‐site mutations (332G‐C, V74L), frameshifts, and M1T—promote expression of the shorter GATA1 isoform, lacking the N‐terminal transactivation domain (N‐TAD), that may modulate DNA binding [28, 29, 30, 31, 32, 33]. Expression of GATA1s, rather than the full‐length protein, contributes to a spectrum of different anemia, underlies Down syndrome‐associated myeloproliferative disorders (transient abnormal myelopoiesis, DS‐TAM) and myeloid leukemia of Down syndrome (ML‐DS) [32, 33, 34].

DNA‐binding proteins such as GATA1 play a critical role in regulating gene expression by interacting with specific regions in the genome. Mutations in these proteins can disrupt their ability to bind DNA effectively, leading to altered transcriptional regulation and contributing to pathogenesis. Despite their importance, many DNA‐binding proteins remain poorly characterized and we lack comprehensive knowledge of how disease‐associated mutations impact their interactions with DNA. While computational approaches, including innovative AI tools like AlphaFold and AlphaMissense, excel at predicting structure‐disrupting mutations, they fall short in assessing more subtle changes, such as mutations that slightly alter DNA‐binding affinity or shift DNA‐binding profiles [35, 36].

Experimental methods used for studying DNA binding are either low throughput, often requiring extensive recombinant protein production [37, 38], or high throughput but only qualitative [39, 40]. The most commonly used electrophoretic mobility shift assays (EMSA) can provide semi‐quantitative insights into binding properties of multiple mutations. However, these experiments are labor‐intensive and often rely on non‐native conditions [41, 42]. Recombinant production of full‐length TFs can be extremely challenging, as these proteins are difficult to handle, especially when destabilizing pathological mutations are introduced. For this reason, most studies focus only on the DNA‐binding domain of GATA1 for biophysical characterization [37, 38]. However, this approach can be misleading, as essential regions contributing to binding may be missing. Additionally, for mutations located outside the DNA‐binding domain, such as in the case of a short GATA1 variant, this strategy is inadequate. Complementary to these biophysical approaches, ChIP‐seq can identify specific binding sites for a given full‐length protein genome‐wide. However, ChIP‐seq results are influenced by chromatin structure and it may miss the effects of mutations that cause subtle changes in DNA‐binding affinity constants [43, 44]. Therefore, a versatile technique is needed to characterize the intrinsic properties of DNA‐protein interactions to assess how mutations alter these parameters.

In this study, we developed a native holdup (nHU) assay, which enables the measurement of equilibrium binding affinities between synthetic or purified DNA baits and full‐length protein preys directly obtained from cell extracts either as endogenous proteins or as transfected ones [45]. This method enables quantitative determination of apparent equilibrium binding constants directly from cell extracts without protein purification, allowing systematic comparison of DNA‐binding affinities of full‐length TFs under physiologically relevant conditions. We demonstrate that nHU is capable of detecting subtle differences in DNA‐protein interactions across multiple sequence variants, which makes it a powerful technique for precisely assessing the binding affinities of GATA1 mutants and can also be used to identify and simultaneously quantify binding partners of short DNA segments proteome‐wide. We investigated the impact of six disease‐causing mutations in GATA1 on DNA‐binding and found that the GATA1 isoform showed markedly increased affinity, while the R307C mutation reduced the affinity of the protein to segments of the *ATP2B4* erythroid promoter. Integrating data from previous studies, analysis of promoter activation and DNA‐binding capacity reveals the distinct functional impacts of different GATA1 mutations on this promoter segment.

## Results

### Characterization of the functional effects of GATA1 mutations on the *ATP2B4* alternative promoter using luciferase assay

**Fig. 2:**
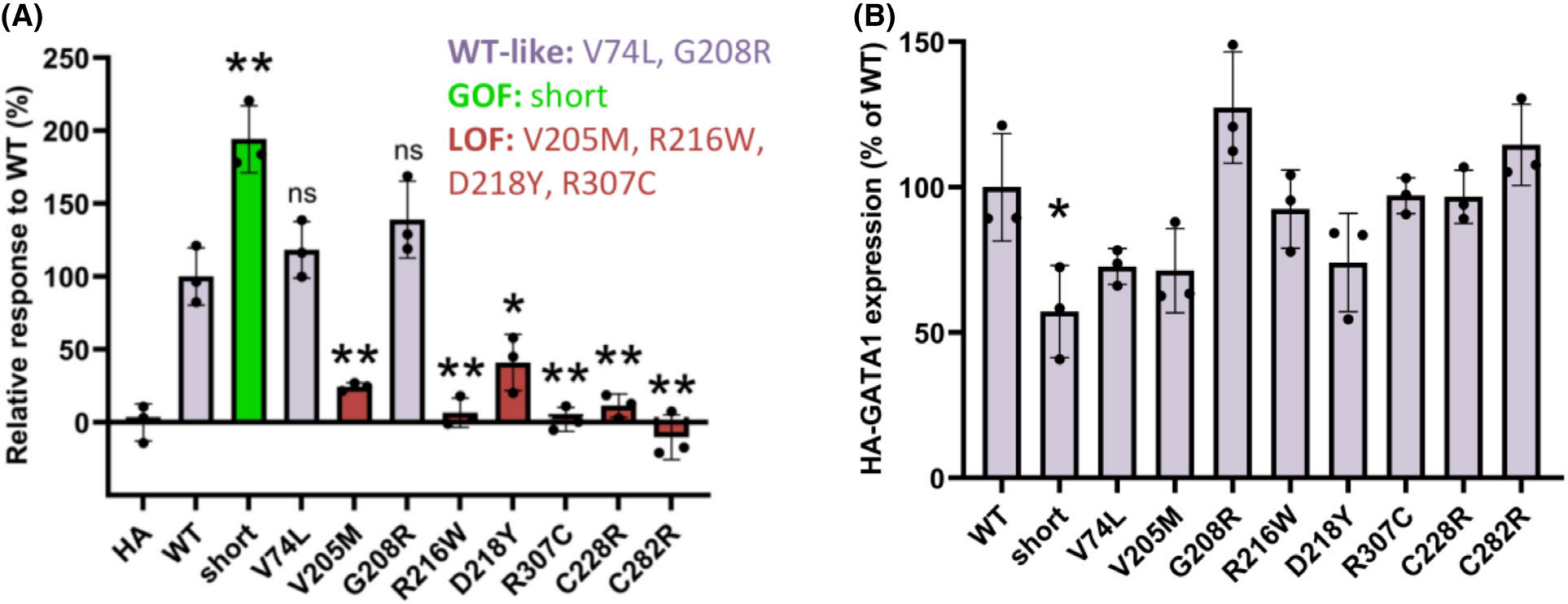
Functional measurement of transcription activation of different mutant forms of GATA1 as measured by luciferase promoter assay using K562 cells and a reporter plasmid containing the full haplotype promoter region of the *ATP2B4* gene. (A) The activity of the different mutant GATA1 proteins in the luciferase assays compared to HA transfected (0%) and HA‐GATA1‐WT (100%) transfected cells. Gray: WT‐like, green: GOF (gain of function), red: LOF (loss of function). (B) The same samples were analyzed by western blot to quantify mutant expression levels, revealing a significantly reduced expression of the short variant. For raw blots and data see Fig. S1 and Data S1. (3 biological and 2 technical replicates, two‐tailed *t*‐test on the average of the values measured for biological replicates, ns: non‐significant, *: *P* < 0.05, **: *P* < 0.01, error bars indicate standard deviation (SD) values).

In order to shed light on the functional effects of different disease‐associated GATA1 mutations on transcription, we performed a luciferase reporter assay in K562 cells. As a reporter construct, we used the alternative erythroid‐specific promoter of the *ATP2B4* gene followed by the Firefly luciferase [11, 17]. We used the haplotype‐containing reporter plasmid with eight predicted single GATA1 sites along with a HA‐GATA1 construct containing either the full‐length WT or the mutant GATA1 sequences in the luciferase assay (Fig. 2A,B, Data S1). A total of 10 different GATA1 constructs were generated, including: the WT sequence; the short variant (GATA1s, or simply short) lacking the N‐TAD; the V74L, which serves as a control because it does not exist naturally in the full‐length context: the nucleotide substitution introduces a splicing defect that results in exclusive production of the short isoform; NF mutations (V205M, G208R, R216W, D218Y); the R307C mutation, which is located just downstream of the CF; and finally, two artificial mutations disrupting the DNA binding of either the NF (C228R) or the CF (C282R) by eliminating one of the cysteines coordinating the zinc ion.

For the artificial C228R and C282R mutations, we observed a complete loss of function in the luciferase assay, emphasizing the critical role of both zinc fingers in maintaining function (Fig. 2A,B). Similarly, the R216W and R307C mutants showed complete loss of function. Partial loss of function was observed for the V205M and D218Y mutations, which are located within the NF region of the protein. No functional impairments were detected with the artificial V74L and the natural G208R mutants, both of which exhibited luciferase activity comparable to the WT. Interestingly, the short GATA1 variant showed an almost twofold increase in activity compared to WT GATA1 (Fig. 2A). Western blot analysis indicated that cells expressed about half the amount of this variant compared to WT (Fig. 2B), suggesting an approximately fourfold increase in partial activity for this mutant when normalized to GATA1 expression (Figs 2 and S1, and Data S1).

### Proteome‐wide affinity characterization of the *ATP2B4* promoter elements by native holdup

**Fig. 3:**
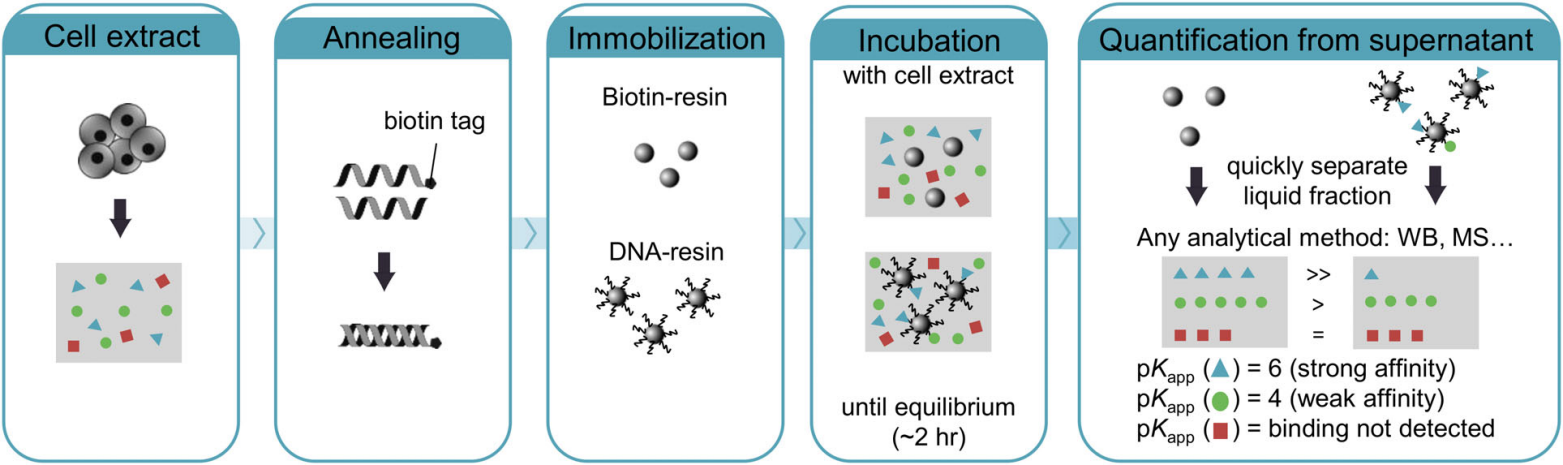
The principles of native holdup using biotinylated oligonucleotide baits. The first step is to produce total cell extracts. Then, annealing is performed using one biotinylated and one non‐biotinylated ssDNA molecule. The annealed dsDNA is immobilized on streptavidin resin along with a control (biotin‐ or scrambled dsDNA‐treated) resin. These saturated resins are then incubated with the cell extract until the equilibrium is reached, after which the supernatants are rapidly separated by centrifugation and quantification is performed from the liquid phase. For quantification, any analytical tool can be used, such as western blot (WB) or mass spectrometry (MS). Finally, the unbound fractions are calculated by comparing the remaining protein fractions for the reaction containing dsDNA to the control reaction and converted to apparent affinity (pK_app_) using the hyperbolic binding equation.

To investigate the DNA binding properties of the studied GATA1 mutants, we decided to adapt the native holdup (nHU) method to DNA‐protein interactions. This technique is advantageous because it is simple, fast, provides quantitative affinity measurements, and does not require protein purification, in contrast to most other assays that evaluate binding constants. Native holdup is a comparative chromatographic approach where a cell extract (analyte) is mixed with a bait‐ or control‐saturated resin and the unbound prey protein fraction is measured from the liquid phase after incubation and rapid separation of the liquid and solid phases (Fig. 3) [45, 46, 47, 48]. From this unbound fraction value and from the estimated bait concentration during the experiment, the affinity of the bait‐prey interaction can be deduced with high precision and accuracy.

**Fig. 4:**
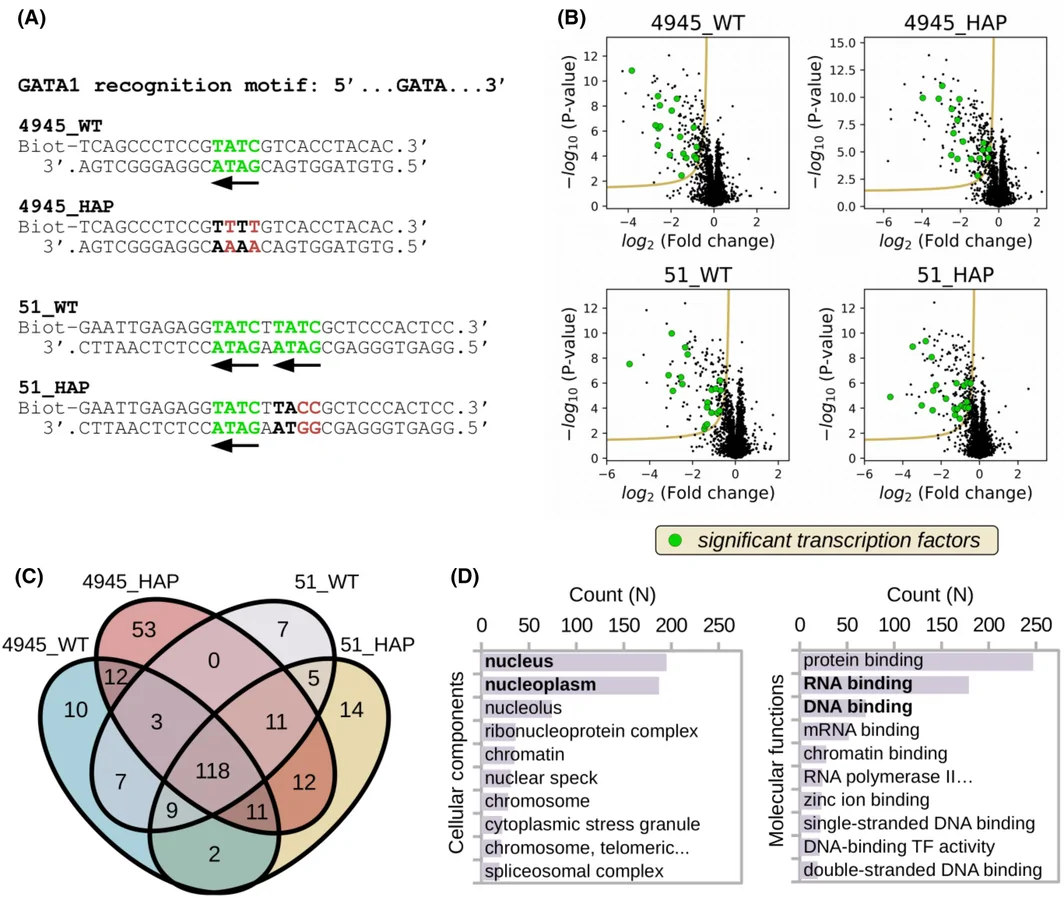
Affinity interactome determination of *ATP2B4* erythroid‐specific alternative promoter elements measured by mass spectrometry‐coupled nHU on K562 cell extracts. (A) Oligonucleotides used in the native holdup assay. Both WT and haplotype (HAP) sequences were used for regions 4945 and 51. For 4945, the single GATA sequence (bold) is abolished by the haplotype, whereas for the tandem region 51 (bold green), one GATA site is eliminated by the haplotype. Bold: GATA recognition sequences (direction indicated with arrows), green: WT sequence, red: haplotype sequence variations. (NCBI GeneID: 439, manual alignment) (B) Volcano plots of four experiments with the four different baits (*n* = 3, injection replicates). Transcription factors are highlighted in green. For further data see Data S2. (C) Overlap of the interaction partners of the different baits, visualized with a Venn diagram (hyperbolic significance threshold was determined at 2σ). Surprisingly, most of the partners found were detectable with all four baits. (D) Gene ontology enrichment was performed using the DAVID tool [49] on the proteins that were binding to at least one DNA bait. Most of the partners detected are general nucleic acid‐binding proteins that are located in the nucleus. For further data see Data S3.

In order to validate the applicability of the native holdup assay for DNA‐protein interactions, we investigated the affinity interactomes of the different *ATP2B4* erythroid‐specific alternative promoter elements by native holdup coupled to mass spectrometry (nHU‐MS, Fig. 4). In previous studies, we performed the assay at relatively high bait concentrations to capture weak and transient protein–protein interactions [45]. However, here we had to consider the nature of nucleic acid‐protein interactions which typically have a higher affinity than protein–protein interactions and these interactions greatly depend on ionic strength. For these reasons, we performed the nHU experiments at a lower estimated bait concentration (5 μm) using a more dilute cell extract (1 mg·mL^−1^). We optimized cell lysis conditions for capturing nucleic acid‐protein interactions by the addition of Mg^2+^ and K^+^ ions and by slightly lowering the ionic strength of the lysis buffer.

We have previously identified two SNP‐containing sites in the alternative promoter region of the *ATP2B4* gene, which had functional impact on transcription, referred to here as 4945 and 51 (abbreviations for SNP identification codes rs10751449/rs10736845 and rs10751451). They contain single and tandem GATA1 recognition sites altered by the haplotype [11]. We used the wild‐type (WT) and haplotype‐containing (HAP) double‐stranded (ds) DNA constructs for the 4945 and the 51 fragments (26‐mer and 31‐mer, respectively) that we hybridized before the nHU experiment (Fig. 4A). To capture the interactions in erythroid type cells, K562 cell extracts were prepared. We chose to use total cell extracts rather than nuclear extracts. This was based on the consideration that the depth of standard MS measurements should be sufficient to detect most relevant nuclear proteins in total cell extracts, while avoiding additional variability due to the nuclear extraction procedure. Apart from these minor modifications, we followed the original nHU protocol with biotinylated‐dsDNA fragments as baits against biotin controls (Fig. 3). After incubating the bait‐saturated resins with cell extracts until equilibrium was reached, we collected the supernatant and analyzed it using routine label‐free quantitative mass spectrometry. In these total proteomic measurements, we managed to quantify the relative amounts of 4560 proteins (Fig. 4B and Data S2). Binding threshold was set as discussed in detail before. Briefly, a hyperbolic threshold was used where significance had to reach a probability of at least 0.05, calculated by a two‐tailed unpaired *t*‐test and prey concentration change needed to be greater than 95% of the measured changes (> 2σ) [45]. Based on this threshold, 274 proteins showed significant binding to at least a single bait (Data S2).

### GATA1 is a sequence‐specific partner of the *ATP2B4* promoter elements measured by native holdup

We detected a surprisingly high number of interaction partners for such short nucleic acid segments. A possible explanation is that nHU can capture indirect interactions. In our previous studies, when large molecular machines were captured, all their subunits were identified as interaction partners [45]. However, of the interactions identified, only a few large complexes have been found, such as the RNA exosome complex, the RNA polymerase III complex, or components of the spliceosome covering only a small fraction of all detected partners (Data S2). Additionally, the different dsDNA‐bait fragments showed remarkably similar interactome in qualitative terms. Among the 274 partners, 118 (43%) were identified as significant partners with all four dsDNA baits, while 152 (55%) and 190 (69%) proteins were identified as partners with three and two baits, respectively (Fig. 4C).

In order to interpret the results of the nHU‐MS experiments, we used the DAVID tool to perform Gene Ontology enrichment analysis for cellular compartments and molecular functions (Fig. 4D and Data S3) [49]. Considering all 274 partners, we have found a great enrichment of proteins localized in the nucleus (*N* = 190; *P* = 2.0 × 10^−44^) or more precisely in the nucleoplasm (*N* = 183; *P* = 1.4 × 10^−67^). These partners were also greatly enriched with nucleic acid‐binding proteins. However, surprisingly, we identified more RNA binding proteins (*N* = 177; *P* = 5.0 × 10^−129^) than DNA binding proteins (*N* = 73; *P* = 7.4 × 10^−24^). Looking more closely at the 118 proteins that we identified as partners with all dsDNA baits, we also found that nuclear proteins (*N* = 89; *P* = 5.4 × 10^−25^), RNA binding proteins (*N* = 79; *P* = 1.1 × 10^−58^), and DNA binding proteins (*N* = 37; *P* = 9.1 × 10^−15^) were highly enriched. Not surprisingly, we also identified many general DNA‐binding proteins among the partners, such as multiple linker histone variants. Therefore, we concluded that the affinity interactomes of nucleic acid baits can be characterized exhaustively by simultaneously measuring sequence‐specific and sequence‐independent interactions using nHU.

Sequence‐independent interactions are general properties of nucleic acids, related to their net charge and conserved topology. In sequence‐independent interactions, molecular contacts are mostly made with the phosphate backbone and the contribution of the nucleic acid sequence is marginal. Focusing only on TFs [50], we identified 32 TFs among the 274 proteins showing binding to at least one of the dsDNA baits (accounting for 11.7%, Data S1). This is apparently a slightly higher ratio than the general occurrence of TFs in the entire genome (1639 TFs among the 20 428 human proteins, accounting for 8.0%) and a much higher ratio than the measured occurrence of TFs in the explored portion of the erythroid proteome of K562 cells (141 detected TFs out of 4560 detected proteins, accounting for 3.1%). However, even TFs can bind indirectly, or in a sequence‐independent manner, similarly to other nucleic acid‐binding proteins discussed above. To identify the TFs that bind in a sequence‐specific way, we exploited the quantitative aspect of the nHU assay by comparing the measured apparent affinities of the wild‐type and haplotype dsDNA baits. By using an unbiased approach, we expected either a gain of affinity or a loss of affinity upon the SNPs. Additionally, since both sites similarly contribute to the regulation of *ATP2B4* gene expression, we assumed that the same interaction partners would have similar effects on both sites. Only a few TFs showed increased affinity for both haplotype baits, such as LIN28B or ATF2, but these showed minor changes in affinity rather than large perturbations (Table 1). Multiple TFs showed decreased affinities to the 51 site, such as ZNF24 or HOXB4, but these proteins did not show detectable binding to the 4945 site. Considering the 4945 segment, the interaction partner showing the highest down‐regulation in affinity was GATA1. GATA1 also showed binding to the 51 site that displayed decreased affinity to the haplotype sequence, albeit only a slight change was measured. Nevertheless, upon ranking of all transcription factors based on their cumulative Δp*K*_app_ values of these sites (which is related to ΔΔG), GATA1 showed the highest overall affinity change out of all partners (Table 1). From these observations, we concluded that GATA1 is indeed the sequence‐specific regulator in this region, consistent with previous findings.

**Table 1:** Identification of sequence‐specific binders by calculating cumulative Δp*K*_app_ for transcription factors detected in the mass spectrometry‐coupled nHU experiments. Apparent affinities that belong to interactions below the significance threshold are colored in gray. To calculate the effect of the haplotype, the WT and haplotype (HAP) apparent affinity differences were summed and the measured p*K*_app_ value of the haplotype sequence was subtracted from the measured p*K*_app_ value of the WT sequence. GATA1 is highlighted in bold as it showed the largest negative effect due to the haplotype.

Accession	Protein	Gene	4945_WT	4945_HAP	51_WT	51_HAP	Effect of haplotype
Q6ZN17	Protein lin‐28 homolog B	LIN28B	6.01	6.46	6.14	6.31	Σ∆p*K*_app_ < −0.4
P17544	Cyclic AMP‐dependent transcription factor ATF‐7	ATF7	5.07	5.14	4.84	5.18	
P15336	Cyclic AMP‐dependent transcription factor ATF‐2	ATF2	5.60	5.80	5.12	5.32	|Σ∆p*K*_app_| < 0.4
P16989	Y‐box‐binding protein 3	YBX3	5.66	5.84	5.87	6.08	
Q02447	Transcription factor Sp3	SP3	4.52	4.47	5.01	5.39	
Q96QR8	Transcriptional activator protein Pur‐beta	PURB	4.64	4.93	4.85	4.88	
P67809	Y‐box‐binding protein 1	YBX1	5.46	5.53	5.48	5.67	
P17483	Homeobox protein Hox‐B4	HOXB4	5.01	5.28	5.47	5.46	
P16220	Cyclic AMP‐responsive element‐binding protein 1	CREB1	5.76	5.96	5.36	5.41	
P17535	Transcription factor jun‐D	JUND	5.80	5.92	5.37	5.47	
Q6DD87	Zinc finger protein 787	ZNF787	5.98	5.95	5.91	6.15	
Q00577	Transcriptional activator protein Pur‐alpha	PURA	5.18	5.30	5.20	5.27	
Q8N5P1	Zinc finger CCCH domain‐containing protein 8	ZC3H8	4.92	4.95	5.08	5.10	
P17482	Homeobox protein Hox‐B9	HOXB9	6.01	6.13	6.12	5.95	
P17028	Zinc finger protein 24	ZNF24	5.18	5.39	5.54	5.24	
P85037	Forkhead box protein K1	FOXK1	5.26	5.16	5.10	5.04	
Q96JP5	E3 ubiquitin‐protein ligase ZFP91	ZFP91	5.57	5.36	5.48	5.45	
O95785	Protein Wiz	WIZ	5.17	4.96	5.09	5.06	
Q8N1G0	Zinc finger protein 687	ZNF687	5.20	4.93	5.12	5.10	
Q9P016	Thymocyte nuclear protein 1	THYN1	6.42	6.19	6.77	6.68	
P17096	High mobility group protein HMG‐I/HMG‐Y	HMGA1	5.97	5.84	6.19	5.97	
P49711	Transcriptional repressor CTCF	CTCF	6.05	5.72	5.98	5.94	
Q9NP66	High mobility group protein 20A	HMG20A	4.98	4.87	5.26	4.98	
P49116	Nuclear receptor subfamily 2 group C member 2	NR2C2	5.22	4.89	4.64	4.51	Σ∆p*K*_app_ >0.4
Q9H5H4	Zinc finger protein 768	ZNF768	5.53	5.19	5.25	5.10	
Q06330	Recombining binding protein suppressor of hairless	RBPJ	5.25	4.96	5.51	5.16	
**P15976**	**Erythroid transcription factor**	**GATA1**	**6.00**	**5.27**	**5.96**	**5.88**	

### *Ab ovo* identification of sequence‐specific interactions with nHU

By using an unbiased approach, we could demonstrate that the nHU assay is well‐suited for identifying sequence‐specific interactions. Exploiting clinically relevant variants allowed us to distinguish relevant interactions from sequence‐independent or irrelevant interactions. Yet, this approach could not clearly separate GATA1 from other transcription factors, leaving the possibility that other interaction partners could also contribute to the differential *ATP2B4* gene expression. Moreover, it would have failed to identify GATA1 as the critical partner of the 51 site alone that only showed small apparent affinity change in the single‐point affinity measurement. To overcome these issues, we decided to use scrambled dsDNA baits, instead of free biotin, to saturate the control resin stock. By comparing binders against this control scrambled dsDNA bait, we can identify all sequence‐specific interaction partners of a given oligonucleotide bait, irrespective of mutations or polymorphisms within the DNA fragment.

**Fig. 5:**
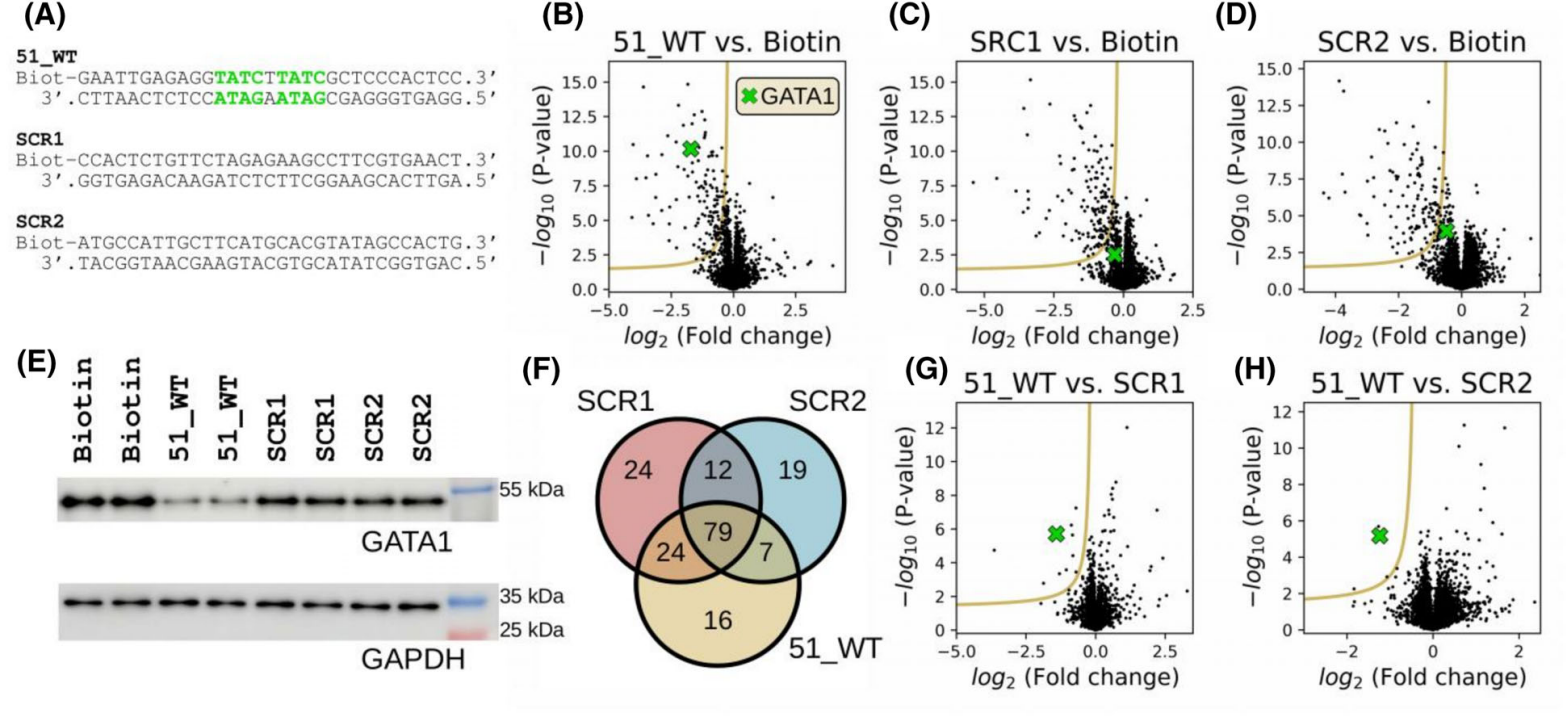
Ab ovo identification of sequence‐specific interactions for a given DNA‐oligonucleotide with nHU coupled to mass spectrometry. (A) Oligonucleotides used in the experiment series. We used the 51_WT sequence and two scrambled versions (SCR1 and SCR2) to find the sequence‐specific recognition partners for the 51_WT bait in an unbiased manner. The GATA sequences are highlighted in green. (NCBI GeneID: 439, manual alignment) (B) Volcano plot visualization of the nHU result with 51_WT sequence compared to the biotin control. (C) Volcano plot visualization of the nHU result with SCR1 sequence compared to the biotin control. (D) Volcano plot visualization of the nHU result with SCR2 sequence compared to the biotin control. All native holdup experiments were carried out with 2 biological replicates and their analytics were done with 3 technical replicates for each biological replicate (*n* = 2 × 3). GATA1 values were determined by western blots from the same samples and highlighted in green in each volcano plots. (E) Western blot analyses of GATA1 levels in the native holdup reactions subjected to mass spectrometry analyses in B–D (2 biological replicates and 3 technical replicates for each biological replicate, *n* = 2 × 3, for full blots see Fig. S2). GAPDH was used as non‐binding loading control. (F) The number of detected significant partners for each oligonucleotide baits visualized with a Venn diagram. Significance was determined based on hyperbolic significance threshold at 2σ. (G) Volcano plot visualization of the native holdup results with the 51_WT bait compared to SCR1 bait. (H) Volcano plot visualization of the native holdup results with the 51_WT bait compared to SCR2 bait. In both cases, GATA1 (green) has been identified as a significant sequence‐specific binder (at 2σ). For further data see Data S4. For all mass spectrometry measurements, injections were performed in triplicate (*n* = 3).

Thus, we repeated the nHU‐MS experiments with the 51 WT fragment but using two additional scrambled DNA fragments as controls (Fig. 5A–D, Data S4). However, the MS runs that were performed for this particular experiment turned out to detect a lower number of proteins in the extract, and GATA1 was among the undetected ones. For this reason, we performed western blot experiments using the same samples that we also measured with proteomics and with the same experimental strategy (same number of replicates, same statistical analysis) to quantify GATA1 protein specifically and included these values in the volcano plots (Fig. 5E–H and Fig. S2). Again, we detected a high number of interactors for all the three DNA probes compared to the biotin‐saturated resin controls, but only in the case of 51 WT sequence we detected GATA1 as a binding partner (Fig. 5B–D). In total, we had 79 partners that were found to interact with all three dsDNA baits and 43 further proteins found to bind two of the dsDNA baits (Fig. 5F). Therefore, just as in the first experiment, the measured interactomes of the different dsDNA baits were similar and we only found 16 specific binding partners for the 51 sequence.

When we used the SCR1 or SCR2 separately as control samples, we only detected 10 or 4 statistically significant binding partners, respectively, for the 51 WT sequence (Fig. 5G,H and Data S4). Among them, only 3 were common in both comparisons, namely TMEM258, CNBP and GATA1. TMEM258 is a transmembrane protein and is therefore spatially separated from DNA and can only interact with the bait due to solubilization by detergent treatment during lysis. CNBP is a CCHC‐type seven zinc finger nucleic acid‐binding protein that preferentially binds single‐stranded oligonucleotides. The predicted binding motif consensus is somewhat consistent with our 51 WT sequence [51, 52]. Due to this, we believe that our annealing was not completely efficient, or considering that the collective electrostatic binding of seven zinc fingers may increase the overall affinity to DNA due to avidity, the protein may appear as a sequence‐specific binder in this study. Finally, GATA1 was the only protein that is a transcription factor and also binds to dsDNA. Thus, we were able to identify the relevant transcription factor that binds to our target sequence even without the use of sequence‐disrupting mutations. Importantly, GATA1 shows minimal binding to scrambled DNA sequences, indicating that it does not bind strongly to DNA baits in a sequence‐independent manner under native conditions. Therefore, most of the observed interactions between the various *ATP2B4* baits and GATA1 detected in the nHU‐MS experiment are driven by sequence‐specific interactions.

### Biochemical characterization of the interactions between the *ATP2B4* promoter elements and GATA1

**Fig. 6:**
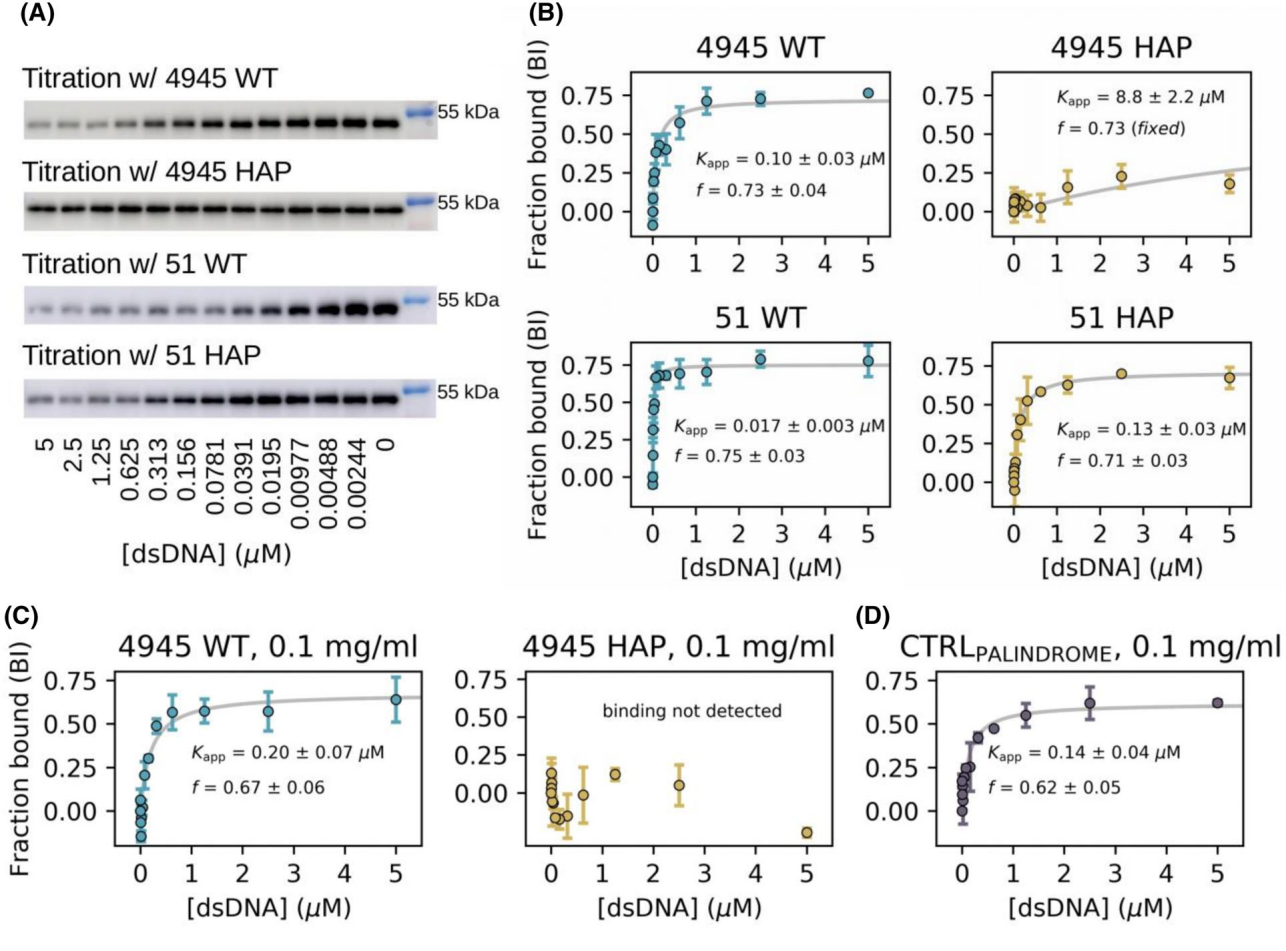
Biochemical characterization of DNA binding of GATA1 to the WT and haplotype‐carrying *ATP2B4* promoter elements using nHU titration coupled to western blot on K562 cell extracts. (A) Western blot results of the titrations with the different baits showed in Fig. 3A (*n* = 2). (B) Hyperbolic binding curves were fitted on fraction bound values (BI) to each data showed in A to calculate apparent affinities (K_app_). 25–30% of the GATA1 protein found in the cell extract was inactive (f: active fraction, *n* = 2). (C) The results of the repeated titration experiments with diluted cell extracts (0.1 mg·mL^−1^, *n* = 2). Left: 4945_WT bait, right: 4945 haplotype bait. (D) Validation of measured affinity values and the estimated bait concentrations of the native holdup experiments, by a native holdup titration using a previously characterized palindromic GATA1 bait (*n* = 2) [37]. Error bars indicate standard deviation (SD) values on each figures. For full blots see Fig. S3.

Although the single‐point nHU experiment used in combination with mass spectrometry provides a proteome‐wide estimation of the affinity interactome of a given oligonucleotide, it only provides a first estimate of affinity constants, based on the assumption that each prey species is fully active for binding, and follows a simple binding mechanism. To address this issue, we performed titration nHU experiments and monitored GATA1 depletion from the liquid phase with western blot (Fig. 6A and Fig. S3). We found that both WT *ATP2B4* fragments displayed simple binding mechanism (matching a hyperbolic binding equation) but they also showed a partial GATA1 binding activity of about 75%, meaning that 25% of the endogenous GATA1 is unable to interact with the bait DNA fragments even at high bait concentrations (Fig. 6B). We also found that the fragment 51, that contains two consecutive GATA motifs in tandem orientation, mediates higher affinity interactions with GATA1 with a dissociation constant of 17 nm, while the fragment 4945, that contains a single GATA motif, mediates a somewhat weaker affinity interaction with GATA1 with a dissociation constant of 100 nm (Fig. 6B). By repeating the titration experiment with the haplotype sequences, we found that both promoter fragments displayed a weaker interaction. The haplotypic version of fragment 51, which still contains a single GATA motif, showed a dissociation constant of 130 nm with GATA1, which is about 10‐fold weaker than that of the WT 51 sequence, and similar to the affinity measured for WT 4945, which also contains a single motif. The haplotype version of fragment 4945, that do not contain any GATA motif, displayed only a marginal interaction, approximately 100‐times weaker than the WT sequence (Fig. 6B), possibly due to marginal sequence‐independent binding. Thus, by performing native holdup titration experiments, we can precisely determine the equilibrium binding constants of DNA‐protein interactions and detect even small differences in affinities, which is essential for characterizing the effects of transcription factor mutations.

Under the holdup condition, a large bait excess has to be guaranteed, ensuring that the measured unbound fraction values of the preys are not affected by their total concentrations in the extract. Since DNA baits may recruit a wide range of potential preys including highly abundant ones such as histones, this may lead to occupation of a significant portion of the resin‐immobilized bait. This might cause competition between different prey molecules for the limiting amounts of baits, affecting the outcomes of nHU experiments. To test whether this effect might be present, we repeated the nHU titration experiment with the 4945 WT sequence but using 10‐fold diluted (0.1 mg·mL^−1^ total protein concentration) K562 cell extract (Fig. 6C, Fig. S3). No evidence of this inhibitory effect was found, as similar affinities were obtained as before (K_app_ = 200 nm). Additionally, we use empirically estimated bait concentrations based on prior studies of protein–protein interactions. However, the strong charge of nucleic acids can influence the bait immobilization step, potentially resulting in lower bait concentration estimates than expected, even though we used higher ionic strength during resin immobilization to minimize this effect. To verify our measured affinities, we performed a nHU titration experiment using a palindromic DNA fragment that has been characterized before *in vitro*, using purified recombinant GATA1 protein with surface plasmon resonance [37]. In the nHU assay we obtained a dissociation constant (140 nm) very similar to that previously measured with SPR (150 nm), confirming that our bait concentration estimation is in the appropriate range (Fig. 6D and Fig. S3).

### Determination of the effect of disease‐associated GATA1 mutations on DNA binding properties by DNA‐nHU

**Fig. 7:**
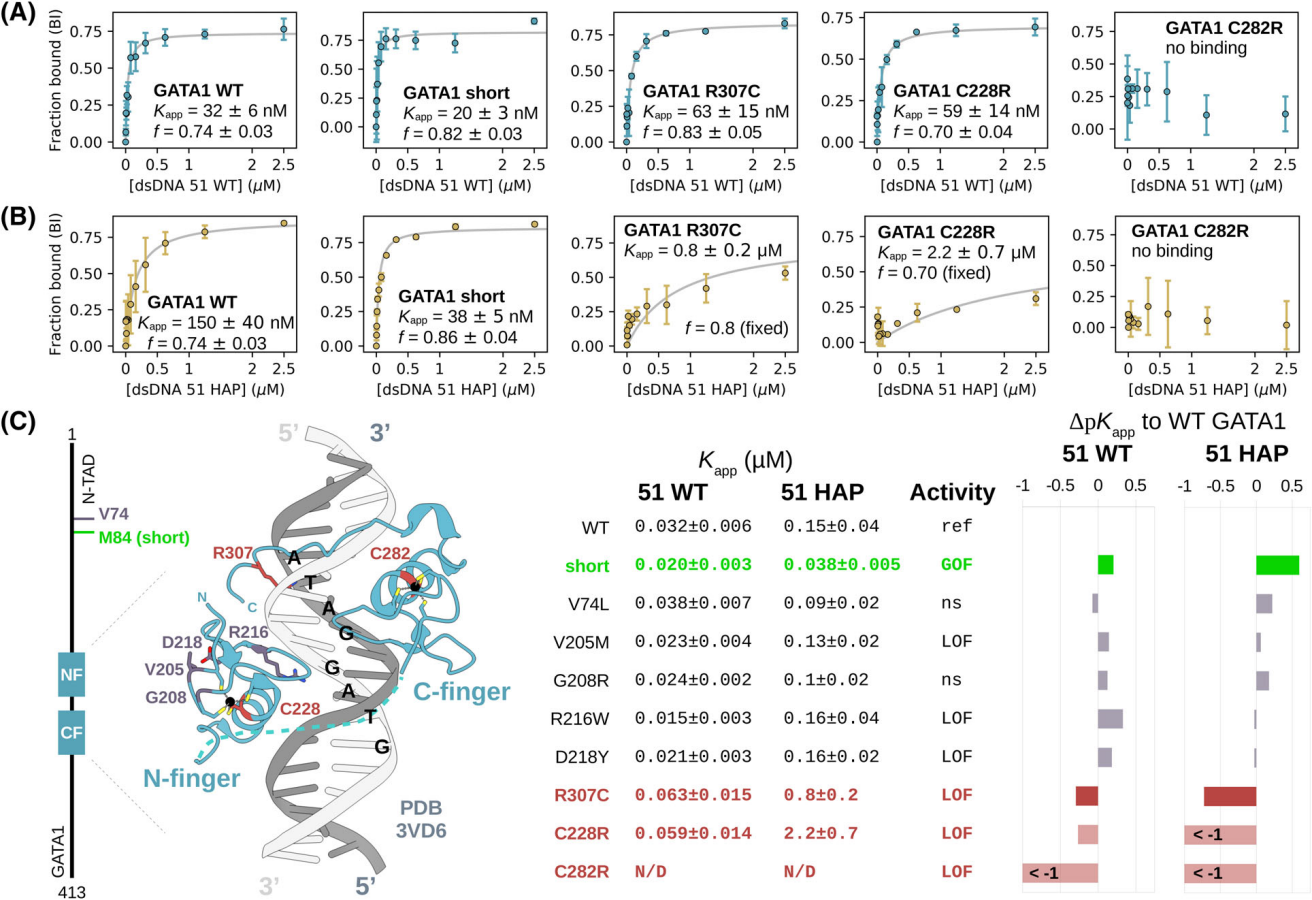
The results of nHU titrations coupled to WB with the different mutant forms of GATA1 using the 51_WT and haplotype sequences and the summary of the effects of different mutations. (A) The hyperbolic binding equations were fitted to the native holdup titration data and plotted. The 51_WT oligonucleotide was used as archetypal tandem GATA motif. We used HEK293T cell extracts previously transfected with plasmids containing different mutant forms of GATA1 protein. For the western blot images and the results with other mutants see Fig. S6. (B) Hyperbolic binding curves on titrations with the 51_HAP (haplotype) sequence as archetypical single GATA motif (performed as in A). Error bars indicate standard deviation (SD) values (*n* = 2). For the western blot images and the results with other mutants see Fig. S7. (C) Summary of the results of the nHU titrations. Left: The different mutations in GATA1 are plotted on the structure of the protein (visualization: ChimeraX, PDB: 3VD6) [38]. Right: Kapp values calculated from native holdup titrations with the 51_WT and 51_HAP sequences (*n* = 2, mean ± SD). Activity: transcription activation measured in the luciferase assay for the different mutant GATA1 proteins (ref: reference activity (WT), ns: non‐significantly different (WT‐like), GOF: gain of function, LOF: loss of function). For all blots and fitted plots see Fig. S5 (51_WT) and S6 (51_HAP).

To analyze the effect of GATA1 mutations on DNA binding, we ectopically expressed these mutants with HA‐tag in order to be able to detect all the different mutant forms of the protein (Fig. S4). Importantly, it has been shown before that N‐terminal tagging does not affect its DNA binding activity [37]. We used the same HA‐GATA1 constructs containing the different mutant versions of the protein than previously in the luciferase assay. We chose HEK293T cell line to express them because it lacks detectable levels of endogenous GATA1 protein or mRNA, preventing any interference with our transiently expressed protein [17]. HEK293T cells expressed all selected GATA1 mutants comparably to the wild‐type protein (Fig. S4). As baits in nHU experiments, we selected the WT and the haplotype 51 dsDNA fragments as archetypical tandem, or single GATA motifs (Fig. 4A). We performed 12‐point nHU titration experiments similarly as shown before and analyzed the results by western blot against the HA‐tag (Fig. 7A,B and Figs S5A,B and S6A,B). Importantly, the ectopically expressed HA‐tagged WT GATA1 displayed nearly indistinguishable intrinsic binding affinities towards both tested baits compared to the naturally occurring (untagged) WT GATA1 found in K562 extracts (Fig. 6A,B and Fig. 7A,B).

The artificial mutation affecting the C‐terminal zinc finger (CF) resulted in complete loss of activity on both DNA fragments, while the N‐terminal zinc finger (NF) mutation reduced the affinity by twofold for the tandem sequence and approximately 15‐fold for the single GATA sequence. This observation is in harmony with previous findings that the NF alone has a very weak affinity for DNA and likely plays a stabilizing and modulatory role after the CF binds to the DNA [38]. Notably, the single GATA site provided much greater differences between the mutant forms of GATA1 compared to the tandem site, which may be due to synergistic effects between neighboring GATA sites. Most pathological GATA1 mutations did not cause major perturbation in DNA binding in a consistent manner for both sites, including the loss of function V205M, R216W, and D218Y mutations. These data suggest that reduced biological activity originates from other mechanisms than reduced DNA binding. Most importantly, we found that two disease‐associated mutations impact DNA binding (Fig. 7A–C). The R307C mutation displayed a twofold decrease in affinity for the tandem GATA motif and approximately a 5.3‐fold decrease in affinity for the single GATA motif. In contrast, the short isoform GATA1s exhibited a 1.6‐fold increased affinity for the tandem and a fourfold increase for the single GATA site, explaining the increased biological activity observed in the luciferase assay.

**Fig. 8:**
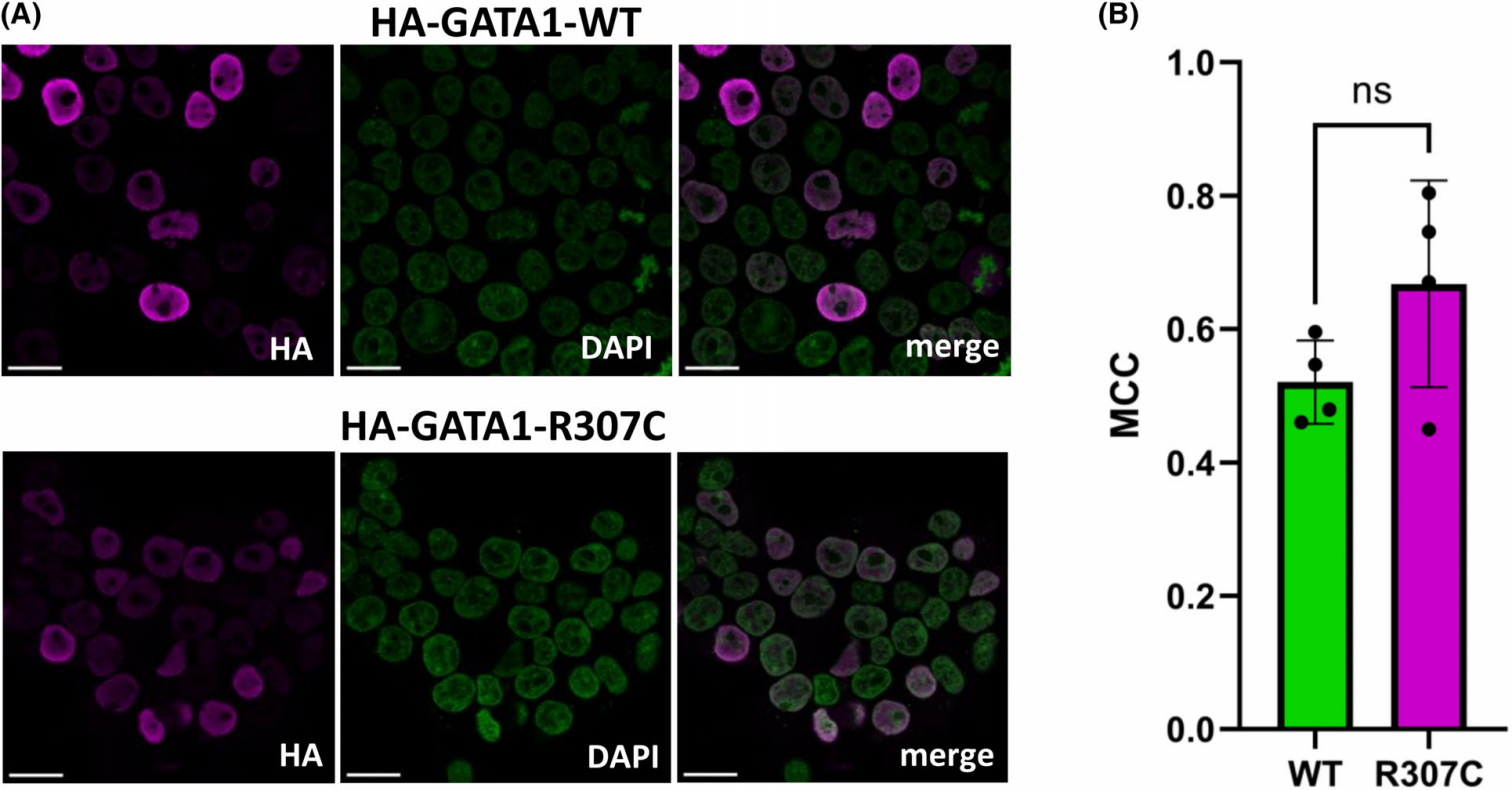
Nuclear localization of the R307C GATA1 protein. (A) Localization of the HA‐tagged WT and R307C mutant GATA1 proteins in transfected HEK293T cells (scale bar: 20 μm). Magenta: HA‐GATA1, green: DAPI. For further images see Fig. S7. (B) Colocalization of HA‐GATA1 with DAPI was determined by Manders' correlation coefficient (MCC) tM1 (the fraction of HA‐GATA1 in compartments containing DAPI). Error bars indicate standard deviation (SD) values. Statistical significance was determined by two‐tailed *t*‐test (ns: non‐significant, *n* = 4, on full images excluding dividing cells).

It has been suggested before, that the R307C mutation can cause mis‐localization, possibly by the disruption of a putative nuclear localization signal [27]. To investigate the possibility that mis‐localization could play a role in the reduced activity of the protein in the luciferase assay, we examined the colocalization of HA‐GATA1 variants with DAPI staining in transfected HEK293T cells (Fig. 8A and Fig. S7). In our assay, all GATA1 variants localized to the nucleus and we did not observe any mis‐localization of the different mutant proteins (Fig. 8B). We concluded that the previously observed slight mis‐localization might result from the disruption of the DNA‐binding capacity of the protein caused by the mutation, and most likely this segment of the protein is not a nuclear localization signal but instead plays a role in DNA binding.

## Discussion

### Functional insight into GATA1 mutations revealed by native holdup

The DNA binding information from nHU experiments helps us to better understand functional differences observed for different mutations localized in the *GATA1* gene. Out of the four NF mutations investigated here, three (V205M, R216W and R218Y) caused loss of function in the luciferase reporter assay using the alternative promoter of the *ATP2B4* gene, but we could not measure altered DNA binding for these variants on the short DNA fragments of this promoter (Fig. 7C). These mutations structurally localize to the same surface of the protein, and it has been previously described that they disrupt the binding of important co‐activators, such as FOG1, LMO2 or TAL1, leading to reduced transcriptional activation by GATA1 indirectly [6, 18, 39, 42]. Of note, one of the studies also found that mutations of these positions caused different degrees of loss of function, due to the differences in structural incompatibility of the different GATA1 protein–protein interaction interfaces [39].

In contrast to the NF mutations, two natural variants directly affected DNA binding: the R307C variant and the short isoform. The R307C variant causes a decrease in affinity which manifests in a reduced cellular activity. This aligns well with the artificial zinc finger mutations that abolish the DNA binding, causing a complete loss of function. It has previously been suggested that mutation in this position may abolish a nuclear localization signal, but our results indicate that it is more likely that the DNA‐binding capacity of this mutant is impaired, which may cause the slight mis‐localization observed before [27]. Indeed, structurally, it has been previously described that this arginine is located in the extension of the CF, protruding deep into the minor groove, and most probably contributes to the DNA binding [38] (Fig. 7C). It has also been proposed that phosphorylation at Ser310 may influence the protein's function, which is disrupted by the R307 mutation [53]. Further analysis is needed to understand the interplay between the protein's phosphorylation states and the DNA‐binding affinity.

In contrast, increased DNA binding affinity was observed for the short GATA1 variant, which also showed enhanced cellular activity in the luciferase assay with the *ATP2B4* erythroid promoter. The observed allosteric effect on DNA binding can be explained by the removal of a potential inhibitory control in the N‐TAD of GATA1 when binding to certain regions in the genome. This mutation has been shown to specifically disrupt chromatin occupancy and H3K27 methylation pattern at erythroid‐specific genes and lineage‐specific effects on maintaining progenitors [28, 30, 54], indicating that, despite the stronger binding observed to short DNA fragments in this study, it may affect the transcriptional activity differently across various genomic sites. Further analysis with DNA‐nHU using different GATA1 recognition sites could reveal the differential binding for different sequences. Additionally, standard protein‐nHU could be used to reveal the quantitative interactome of N‐TAD, using different cell extracts to identify specific regulatory proteins in distinct cellular milieu. Overall, by integrating previous protein–protein interaction results with comparative functional activity data and a systematic comparison of DNA binding activities measured by nHU, our study provides new insights into the molecular mechanisms underlying the disease‐associated mutations in GATA1.

Previous studies using the mouse G1E/G1ME complementation systems, mouse models or hematopoietic progenitors carrying the mutations have similarly shown that different GATA1 mutations lead to varying degrees of functional impairment [18, 22, 28, 30, 37, 39, 54]. Some mutations probably affect DNA binding directly [22, 37, 39], while others disrupt interactions with cofactors like FOG1 [18, 39], LMO2 [39], the TAL1 complex [39], or unknown partners. The impact on transcription also depends on the genomic context, as GATA1 likely requires different cofactors at different binding sites [28, 39, 54]. For example, the complexes involved in gene activation may differ from those needed for repression. Adding further to the complexity, it has been shown that GATA1 mutations can dysregulate blood cell development in a stage‐specific manner, as fetal and adult blood cells originate from distinct progenitor cell populations [55]. Nevertheless, by clarifying how these mutations alter DNA‐binding affinity of the full‐length human GATA1 and exploring its direct link to transcriptional activity at a given site, we may better interpret their effects in functional assays and understand the broader consequences for transcriptional regulation.

### Opening new frontiers in transcription factor research with native holdup

Using the erythroid‐specific promoter segments of the *ATP2B4* gene as a model system, we investigated the DNA binding properties of GATA1 mutants transiently expressed in mammalian cells. Our results demonstrate that native holdup (nHU) is a versatile method to quantitatively measure DNA binding affinities of TFs without requiring protein purification, making this an ideal approach for systematically studying TF mutations. However, we observed some technical limitations, which warrant discussion. For protein partner identification of short DNA sequences, we used the native holdup coupled to mass spectrometry. As with any mass spectrometry‐based method, this assay depends heavily on the quality and depth of the mass spectrometry measurements. For instance, in our second experimental series using scrambled oligonucleotides, we obtained cleaner data but experienced reduced measurement depth, leading to the loss of GATA1 detection. Nevertheless, ongoing advancements in mass spectrometry equipment, software, and accessibility within the research community hold promise for improving the reliability of this method in the future. If the binding partner of a DNA segment has already been identified, nHU coupled with another direct analytical tool, such as western blot, can be used to get a more precise quantification of the binding. For this, even tag‐specific antibodies can be used allowing the characterization of proteins that are challenging to quantify with specific antibodies or engineered proteins that are introduced in the extract by genetic approaches. Another potential limitation is the presence of a high fraction of inactive DNA‐binding protein in the cell extract. In single‐point measurements, this can impact the apparent binding affinity, while in titration experiments, it may complicate the quantification of affinities. To mitigate this issue, it is essential to handle cells and extracts carefully to maximize the recovery of the most active protein fraction. Finally, this method can capture both direct and indirect interactions, but only those indirect interactions are detected that have extremely high affinities and close to stoichiometric expression levels with their directly bound partners. When these conditions are not satisfied, the DNA‐binding setup that we performed here may fail to distinguish mutations, including pathogenic ones, that alter the protein interactome of GATA1 without altering its DNA‐binding affinity. This was possibly the case for mutations V205M, R216W, and D218Y. Although further nHU experiments could be used to explore the affinity interactomes of regulators of GATA1‐NF or N‐TAD regions, they are beyond the scope of this study.

Experimentally, researchers have traditionally used pulldown‐based assays to identify DNA‐protein interactions. However, the dynamic nature of many interactions means that the partners may dissociate during the washing steps, leading to incomplete or misleading results. Our native holdup (nHU) assay addresses these challenges by enabling the identification of DNA‐binding partners without the need for washing steps, making this method ideal for capturing even transient interactions. This method offers a robust and simple way to systematically investigate the effects of disease‐associated mutations in either the DNA or in the DNA‐binding protein. The nHU assay can not only identify the partner of a given DNA bait, but also provides a quantitative measurement of intrinsic affinity. Such data are typically difficult to obtain and require the production of purified proteins at high quantities. These are not requirements of a nHU assay that can be done with affordable synthetic DNA oligos, a small amount of commercially available resin and simple cell extracts. The results that these assays provide are comparable to conventional biophysical assays, such as ITC or SPR, or other methods routinely used in the nucleic acid fields, such as mobility shift assays. In the past, multiple studies measured DNA binding to GATA1 using quantitative biophysical approaches, yet only with a few exceptions these studies were performed with isolated zinc fingers of GATA1, containing no or mild, non‐disruptive mutations, often at non‐physiological buffer composition [38, 39]. In the case of nHU, there is no need to purify partner proteins, hence even highly unstable, yet biologically important full‐length proteins that would be not suitable for conventional methods can be studied at near native conditions. In conclusion, nHU offers new perspectives for studying nucleic acid‐protein interactions and due to the simplicity of the assay, it can be effortlessly implemented in any cell biology laboratory.

## Methods

### Cell culturing

K562 cells (ATCC ref. CCL‐243, RRID:CVCL_0004, not authenticated, cell line was a kind gift of Arnauld Poterszman) were grown in RPMI 1640 medium without HEPES (Gibco) completed with 10% fetal calf serum (FCS) and 40 μg·mL^−1^ gentamicin and diluted 1 : 12 every 3rd/4th day. HEK293T cells were authenticated by short tandem repeat (STR) profiling using ATCC Cell Line Authentication Service (Manassas, VA, USA), showing a 100% match to ATCC ref. CRL‐3216 (RRID:CVCL_0063, the authentication report is available upon request), and were grown in Dulbecco's modified Eagle's medium (DMEM, Gibco, glucose: 1 g·L^−1^) completed with 10% FCS and 1% Penicillin–Streptomycin (Gibco) on CellBIND flasks (Corning, NY, USA), diluted 1 : 10 every 3rd/4th day. All cells were kept at 37 °C and 5% CO_2_. Cells were routinely tested for mycoplasma, and all experiments were performed with mycoplasma‐free cells.

### DNA plasmid constructs and mutagenesis

For the luciferase assay, the pGL3 vector containing the erythroid promoter region of the *ATP2B4* gene with the haplotype was created before (pGL3‐ATP2B4‐H1st plasmid, haplotype carrying version) [17]. For the GATA1 mammalian plasmid, the sequence of GATA1 was cloned from the pIRES2‐GATA1‐GFP plasmid [17] into the pCI standard mammalian vector containing a HA‐tag for N‐terminal tagging with standard restriction cloning.

The different mutant forms of GATA1 were created by using standard site‐directed mutagenesis on the pCI‐HA‐GATA1‐WT plasmid with Platinum SuperFi II polymerase (Thermo Fisher Scientific, Bremen, Germany). Successful mutagenesis was verified by Sanger sequencing (Eurofins Genomics Germany GmbH, Ebersberg, Germany).

### Transfections

For the luciferase reporter assay, 1 × 10^5^ K562 cells/well were seeded the day before the transfection on 24‐well plates. The next day, cells were transfected with the pGL3‐ATP2B4‐H1st‐haplo and pCI‐HA‐GATA1 WT and mutation carrying plasmid vectors using JetPRIME reagent (Polyplus), according to the manufacturer's recommendations using 0.35 μg pGL3 and 0.15 μg pCI‐GATA1 vector DNA and 1 μL JetPRIME reagent/well.

For the cell extract preparation of HA‐GATA1 WT and mutants expressing HEK293T cells, the cells were seeded on poly‐D‐lysine (ref. P6407; Sigma‐Aldrich, St. Louis, MO, USA) treated 100 mm cell culture dishes (2 × 10^6^ cells/dish). The next day, the transfection was carried out with JetPRIME reagent (Polyplus, Illkirch‐Graffenstaden, France) according to the manufacturer's recommendations using 5 μg DNA and 20 μL of JetPRIME reagent/well. The medium was replaced with fresh medium after 5 h of treatment with the transfection mixture.

For the immunostaining of HA‐GATA1 expressing cells, 24‐well plates with coverslips were poly‐D‐lysine treated (ref. P6407; Sigma‐Aldrich), and HEK293T cells were seeded on the plates the day before the transfection (1 × 10^5^ cells/well). The transfection was carried out with JetPRIME reagent (Polyplus) according to the manufacturer's recommendations using 0.5 μg DNA and 1 μL of JetPRIME reagent/well. The medium was replaced with fresh medium after 5 h of treatment with the transfection mixture to enhance cell survival.

### Luciferase reporter assay

24 h after transfection, K562 cells were collected by centrifugation (1 min × 1000 g) and washed once with PBS. Then, 100 μL of 1x passive lysis buffer (5× PLB diluted with UltraPure water, ref. E1941; Promega, Madison, WI, USA) was added to the cells and incubated for 15 min at room temperature under shaking. Lysates were pipetted up and down a few times and 20 μL lysate was transferred into 96‐well white plates (ref. MSSWNFX40; Millipore). Then, 100 μL luciferase assay reagent (ref. E1483; Promega) was added to each well and the firefly luminescence was measured immediately on a PHERAstar (BMG Labtech, Offenburg, Germany) microplate reader by using Lum Plus filter. The remaining cell lysates were kept for expression determination by western blot. In total, 3 biological (transfection) and 2 technical (from the same extract) replicates were performed. For statistics, the technical replicates were averaged and two‐tailed *t*‐tests were performed on these averaged values for each biological replicate (*n* = 3). For statistical analyses, GraphPad Prism 10 software was used. For raw data and calculations, see Data S1.

### Cell extract preparation for nHU assay

Cell extracts were prepared similarly as before [45] except the lysis buffer composition, which was optimized for DNA‐protein interactions: 50 mm Hepes‐KOH (pH 7.5), 100 mm NaCl, 50 mm KCl, 4 mm MgCl_2_, 1% Triton X‐100, 5× cOmplete EDTA‐free protease inhibitor cocktail, 5 mm TCEP, and 10% glycerol. Briefly, K562 cells were collected by centrifugation (1000g × 5 min), quickly washed with PBS, and ice‐cold lysis buffer was added. HEK293T cells were washed once with PBS, lysis buffer was added and cells were collected by scraping. Lysates were sonicated (4 × 20 s with 1‐s‐long pulses on ice) and incubated rotating (4 °C for 30 min). The lysates were centrifuged (12 000 g 4 °C for 20 min), and the supernatant was kept. Total protein concentration was measured by standard Bradford method (ref. 5000006; Bio‐Rad protein assay dye reage, Bio‐Rad Laboratories, Hercules, CA, USA) using a bovine serum albumin (BSA) calibration curve (ref. 160069; MP Biomedicals, Irvine, CA, USA) on a UV‐1700 PharmaSpec spectrophotometer instrument. Extracts were diluted to 1 mg·mL^−1^ concentration and were snap‐frozen in liquid nitrogen and stored at −80 °C.

### Biotinylated DNA oligonucleotides and annealing for nHU

All DNA oligonucleotides were produced by Eurofins Genomics (Eurofins Genomics Germany GmbH, Germany) and were high‐performance liquid chromatography (HPLC) purified (for exact sequences see Table S1). For annealing, the two single‐stranded oligonucleotides were mixed in a 1 : 1.5 forward‐reverse DNA ratio in a 12.5 μm final double‐stranded oligonucleotide concentration in holdup (HU) buffer (50 mm Tris pH 7.5, 300 mm NaCl, 1 mm TCEP, 0.22‐μm filtered). In a pre‐heated thermoblock, the samples were heated up to 95 °C for 5 min, then slowly cooled down within 100 min until they reached room temperature.

In general, 680 μL of 12.5 μm dsDNA were used for the saturation of 25 μL pure streptavidin resin (Streptavidin Sepharose High Performance, Cytiva, Uppsala, Sweden). The estimated bait concentration of the saturated resin is 10 μm once 25 μL pure bait‐saturated streptavidin resin was mixed with 100 μL of cell extract.

### Singe point and titration native holdup

For saturating resin (Streptavidin Sepharose High Performance, Cytiva) with dsDNA or biotin (as control), 25 μL pure resin was mixed with either 680 μL of 12.5 μm dsDNA or 25 μL 1 mm biotin, and additional NaCl was added (at 0.5 m final concentration) to facilitate DNA fixation to the resin. Resin mixtures were incubated for 1 h at room temperature, gently mixing every 15 min. Resins were briefly centrifuged (15 s, 2000 g), the supernatants were removed and the resins were depleted with biotin (10 resin volume, 3 min, holdup buffer supplemented with 100 μm biotin). Then, resins were washed four times (10 resin volume, holdup buffer). To get a bait concentration of 5 μm, these pre‐saturated resins were reconstituted in 6x resin volume of HU buffer and were mixed in a 1 : 1 ratio by adding 12.5 μL dsDNA to 12.5 μL control (biotin) pure resin. Finally, all liquid was removed and cell extracts were added in 1 mg·mL^−1^ concentration in a 1 : 4 pure resin:extract ratio (25 μL pure resin: 100 μL cell extract) and incubated for 2 h at 4 °C with constant gentle agitation. After the incubation ended, the resins were separated from the supernatant by a brief centrifugation (15 s, 2000 g). 75% of the supernatants were quickly pipetted to a separate tube and centrifuged again briefly, and 50% of the original volume of the supernatant was kept in the end to reduce resin contamination in the final samples.

Titration experiments (coupled to western blot) were carried out similarly as before, by mixing control and bait‐saturated resins and keeping the total resin volume constant [45]. Twelve‐point titrations were carried out. For the starting point, the DNA‐saturated and biotin control resins were mixed in 1 : 1 (5 μm starting point) or 1 : 3 (2.5 μm starting point) ratio, typically 20 + 20 μL or 10 + 30 μL, respectively, calculated for the pure resin volume, in 4x resin volume in holdup buffer. Then, a 1 : 1 serial dilution was carried out with the control resin (20 + 20 μL pure resin). The final tube contained only the biotin‐saturated resin as control. The resins were then briefly centrifuged (15 s, 2000 g), the supernatant was removed and cell lysates were added at a concentration of 1 or 0.1 mg·mL^−1^, and further procedures were carried out as described for the single‐point experiments (20 μL of resin was mixed with 80 μL of extract to obtain the correct concentration of bait during the incubation). All the experiments were carried out in Protein LoBind tubes (refs. 022431064 and 022431081; Eppendorf, Hamburg, Germany). All titrations were carried out with a minimum of two replicates.

### Sample digestion and mass spectrometry (LC–MS/MS) analysis

MS analyses were performed as described before [45, 56]. Briefly, nHU samples were precipitated with TCA, and the urea‐solubilized, reduced, and alkylated proteins were digested with trypsin at 2 m final urea concentration. Peptide mixtures were then desalted on a C18 spin‐column and dried on Speed‐Vacuum. 100 ng peptide mixtures were analyzed using an Ultimate 3000 nano‐RSLC coupled in line, via a nano‐electrospray ionization source, with an Orbitrap Exploris 480 mass‐spectrometer (Thermo Fisher Scientific, Germany) equipped with a FAIMS (high Field Asymmetric Ion Mobility Spectrometry) module. Data was collected in DDA (data dependent acquisition) mode; proteins were identified by database searching using SequestHT (Thermo Fisher Scientific, Germany) with Proteome Discoverer software (Thermo Fisher Scientific, Germany). Peptides and proteins were filtered with a false discovery rate (FDR) at 1%. Label‐free quantification was based on the extracted ion chromatogram (XIC) intensity of the peptides. All samples were measured in three technical triplicates. The measured XIC intensities were normalized based on median intensities of the entire dataset to correct minor loading differences. For statistical tests and enrichment calculations, not detectable intensity values were treated with an imputation method, where the missing values were replaced by random values similar to 10% of the lowest intensity values present in the entire dataset. Unpaired two‐tailed *t*‐tests, assuming equal variance, were performed on obtained log_2_ XIC intensities. All raw LC–MS/MS data have been deposited to the ProteomeXchange via the PRIDE database with identifier PXD055181. Obtained fold‐change values were converted to apparent affinities using the hyperbolic binding equation, and binding thresholds at 2σ were determined as described before in detail [45].

### Gene ontology enrichment analysis

Gene Ontology enrichment analysis was performed using the DAVID tool with default parameters [49]. Two independent analyses were performed using all the preys that were interacting with at least one bait, or using only those preys that were interacting with all baits, and both analyses were done against the same background (all proteins detected by the proteomic measurements). Only the Cell Component (CC_DIRECT) and Molecular Function (MF_DIRECT) terms were analyzed, and all significant terms are included in Data S3.

### Identification of sequence‐specific binders based on cumulative Δp*K*_app_

To calculate affinity differences between interactions measured with the WT and haplotype‐carrying dsDNA baits, we used the apparent affinities that were converted from the measured fold‐change values. First, we identified all transcription factors among the interaction partners of the dsDNA baits using a recently reported comprehensive transcription factor catalog [50]. Then, we collected the apparent affinities of all these proteins and calculated the overall effect of the haplotype by summing the affinity differences between WT and HAP sequences. For getting the affinity differences, we simply subtracted the measured p*K*_app_ value of the haplotype sequence from the measured p*K*_app_ value of the WT sequence, which is proportional to the ΔΔ*G*_app_ values [56]. For the affinity matrix for WT and haplotype sequences, see Data S3.

### Western blot

Western blots were done similarly as before [45]. The samples were mixed with 4× Laemmli buffer (120 mm Tris/HCl pH 7, 8% SDS, 100 mm dithiothreitol, 32% glycerol, 0.004% bromophenol blue, and 1% β‐mercaptoethanol) in a 3 : 1 ratio. Equal amounts of samples (generally 6 μg) were loaded on 12% acrylamide gels. Transfer was done using a Trans‐Blot Turbo Transfer System and Trans‐Blot Turbo RTA Midi 0.45 μm LF PVDF Transfer Kit (ref. 1704275; Bio‐Rad). After 1 h of blocking in 5% milk in TBS‐Tween (1x Tris‐buffered saline, 0.1% Tween 20), membranes were incubated overnight at 4 °C in primary antibody in 5% milk in TBS‐Tween. The following antibodies and dilutions were used: anti‐GATA1 (1 : 10000, ref. ab181544; Abcam, Cambridge, UK, RRID:AB_2920794), anti–glyceraldehyde‐3‐phosphate dehydrogenase (GAPDH, 1 : 5000, ref. MAB374; Sigma‐Aldrich, RRID:AB_2107445), and anti‐HA.11 epitope tag (1 : 5000, ref. 901501; BioLegend, San Diego, CA, USA, RRID:AB_2565006). Membranes were washed three times with TBS–Tween and incubated at room temperature for 1 h in secondary antibody solution [Jackson ImmunoResearch, West Grove, PA, USA, peroxidase‐conjugated Affinipure goat anti‐mouse (H + L), ref. 115‐035‐146 RRID:AB_2307392 and goat anti‐rabbit (H + L), ref. 111‐035‐003 RRID:AB_2313567] in 5% milk (concentration 1 : 10000). After washing three times with TBS‐Tween, membranes were exposed to chemiluminescent horseradish peroxidase substrate (ref. WBKLS0100; Immobilon, MilliporeSigma, Burlington, MA, USA) and photographed in a docking system (Amersham ImageQuant 800). Densitometry was carried out on raw Tif images by using Fiji imagej 1.54 f. Between different primary antibody labeling, the membranes were either exposed to 15% H_2_O_2_ to remove secondary signal (in the case of different species) or stripped with mild stripping buffer (15 g·L^−1^ glycine, 1 g·L^−1^ SDS, and 1% Tween 20, pH 2.2) to remove primary signal (in the case of same species). For native holdups, GAPDH was used as a non‐binding control, but was not used to normalize GATA1 values as this induces technical errors coming from blotting issues rather than from differences between the samples, as discussed before [45]. In case of inconsistencies in GAPDH values, the blot was repeated.

### Immunostaining of HA‐GATA1‐transfected cells and colocalization analysis

24 h after transfection, HEK293T cells were washed once with PBS and fixed with 4% PFA (ref. 15710; Electron Microscopy Sciences, Hatfield, PA, USA) in PBS for 10 min at room temperature. The cells were then washed three times with PBS and permeabilized with 500 μL/well of 0.2% Triton X‐100 (ref. T8787; Sigma‐Aldrich) in PBS for 10 min at room temperature. After permeabilization, the samples were blocked in blocking buffer [0.05 g mL^−1^ BSA (ref. 160069; MP Biomedicals), 0.1% Triton X‐100 in PBS] for 1 h at room temperature. Then, the cells were incubated overnight with the HA primary antibody (anti‐HA.11 epitope tag, dilution: 1 : 1000, ref. 901501; BioLegend, RRID:AB_2565006) at 4 °C in blocking buffer. The next day, the cells were washed three times with PBS with 10 min incubations each time at room temperature. Then, the cells were incubated with the secondary antibody (Alexa Fluor 594–conjugated anti‐mouse, dilution: 1 : 1000; Invitrogen, Carlsbad, CA, USA, ref. A‐11032, RRID:AB_2534091) in blocking buffer. After three times 10 min PBS washes, the coverslips were mounted to microscopy slides by Vectashield mounting medium with 4′,6‐diamidino‐2‐phenylindole (DAPI) (Vector laboratories, Burlingame, CA). Images were taken using a Leica SP5 confocal microscope (Leica Camera AG, Wetzlar, Germany) with an HCX PL APO 63×/1.40– 0.60 oil objective using excitation at 405 nm (diode) and 594 nm (HeNe laser) and emission at 415–480 and 610–695 nm for DAPI and Alexa 594, respectively. Images were processed by the Fiji imagej 1.54f software. To determine colocalization of HA‐GATA1WT and mutant proteins, Coloc2 plugin was used. For each image, the background was subtracted, and three ROIs of the same size were selected, excluding non‐transfected cells, large background areas, and dividing cells. After ROI selection, the Coloc2 plugin was used with the Costes automated threshold to determine Manders' tM1 (MCC), representing the fraction of HA signal in compartments containing DAPI. The three values obtained for each image were then averaged. In total, four images per GATA1 variant were statistically probed using two‐tailed Student's *t*‐test (*n* = 4).

## Conflicts of interest

The authors declare no conflicts of interest.

## Author contributions

BZ conceptualized the study, designed and performed most of the experiments. BZ and GG designed and performed all the nHU and did all the final data analyses. BM and LN performed the MS experiments and primary data analysis. OM and BS took part in conceptualization and helped in the luciferase assay. GT obtained funding for the experiments. GT and GG supervised the project. BZ and GG wrote the original draft, and all authors reviewed the manuscript.

## Supporting information


**Data S1.** Results of the luciferase assay measurements and the related western blots for expression determination.

**Data S2.** Results of the nHU‐MS experiments.

**Data S3.** Results of the GO enrichment.

**Data S4.** Results of the nHU‐MS experiments of the scrambled DNA fragments.

**Fig. S1.** Western blot images on transfected K562 cell extracts that were used in luciferase assay, to determine the HA‐GATA1 expressions.
**Fig. S2**. Western blot images of single‐point nHU experiments with 51_WT, SCR1 and SCR2 sequences.
**Fig. S3**. Western blot images of titration nHU experiments with the 4945_WT, 4945_HAP, 51_WT, 51_HAP, or with the CTRL_PALINDROME_ sequences on K562 cell extracts either at 1 mg·mL^−1^ total protein concentration (upper panel) or 0.1 mg·mL^−1^ total protein concentration (lower panel).
**Fig. S4**. Validation of transient expressions of the different mutant versions of GATA1 in HEK293T cells by western blot.
**Fig. S5**. Raw western blot images and additional binding curves for GATA1 mutants binding to 51_WT sequence.
**Fig. S6**. Raw western blot images and additional binding curves for GATA1 mutants binding to 51_HAP sequence.
**Fig. S7**. Additional images on the localization determination of different mutant versions of the GATA1.
**Table S1**. Biotinylated oligonucleotides used for native holdup.

## Data Availability

All raw LC–MS/MS data have been deposited to the ProteomeXchange via the PRIDE database with identifier PXD055181. Material requests should be addressed to Boglarka Zambo and Gergo Gogl.
