# Supplementary material for: Quantitative analysis of DNA‐GATA1 binding alterations linked to hematopoietic disorders
Source: FEBS J. 2026 Mar 17;293(17):5149–69. doi: 10.1111/febs.70483 (PMC13534945; doi:10.1111/febs.70483)
Supplement: Supplementary file 1 [file FEBS-293-5149-s004.pdf]

Supporting information legends:

Supplementary Data 1. Results of the luciferase assay measurements and the related Western blots for expression determination. The first sheet contains the luminescence values measured with transfected K562 cells and an *ATP2B4* erythroid-promoter construct. The assay was performed with 3 biological and 2 technical replicates, data were normalized that the average of HA was 0 and the WT was 100 (%). The second sheet contains expression result by densitometry on Western blots, on the same samples as the luminescence was measured. For the Western blot images see Supplementary Figure S3.

Supplementary Data 2. Results of the nHU-MS experiments. The first four sheet contains the measured $\log_2$ fold change values, statistical significances ($-\log_{10}P$), calculated binding intensities, apparent affinities and significance thresholds. In cases where the significance threshold is smaller than the obtained statistical significance of the measurement, the apparent affinity is considered significant and a numerical affinity value is displayed in the p$K_d$sign column. Proteins that are classified as transcription factors are highlighted in the last column. The last tab contains an assembled affinity matrix of all significant affinities.

Supplementary Data 3. Results of the GO enrichment. On the first sheet, an assembled affinity matrix of all significant affinities is shown with the last column showing the number of significant measurements for each prey. GO enrichment analysis was performed for all proteins with >0 values in this column or for the promiscuous proteins for proteins with a value of 4 in this column. The following sheets contain the significant GO terms for cellular compartments or for molecular functions.

Supplementary Data 4. Results of the nHU-MS experiments of the scrambled DNA fragments. Similarly to Data 2, each sheet contains the measured $\log_2$ fold change values, statistical significances ($-\log_{10}P$), calculated binding intensities, apparent affinities and significance thresholds and the significant affinities. The first three sheet contains the measurement of the SCR1, SCR2 and the 51WT baits against a biotin control. The last two sheets contain the measurement of the 51WT bait against the SCR1, or SCR2 controls.

Supplementary Figure S1. Western blot images on transfected K562 cell extracts that were used in luciferase assay, to determine the HA-GATA1 expressions.

Supplementary Figure S2. Western blot images of single point nHU experiments with 51_WT, SCR1 and SCR2 sequences.

Supplementary Figure S3. Western blot images of titration nHU experiments with the 4945_WT, 4945_HAP, 51_WT, 51_HAP, or with the $CTRL_{PALINDROME}$ sequences on K562 cell extracts either at 1 mg/ml total protein concentration (upper panel) or 0.1 mg/ml total protein concentration (lower panel).

Supplementary Figure S4. Validation of transient expressions of the different mutant versions of GATA1 in HEK293T cells by Western blot. The HA antibody recognizes all mutants, but the GATA1 antibody doesn't recognize the short version of GATA1 (GATA1s) as the epitope recognized by the antibody might located at the N-terminal part of the protein.

Supplementary Figure S5. Raw Western-blot images and additional binding curves for GATA1 mutants binding to 51_WT sequence. **A.** Western blot images of nHU titrations with the 51_WT sequence on cell extracts prepared of HA-GATA1 WT and mutant transfected HEK293T cells. **B.** Determined hyperbolic binding curves on titrations with the 51_WT bait and with the mutant GATA1 proteins that are not shown in Figure 6. Error bars indicate standard deviation (SD) values.

Supplementary Figure S6. Raw Western-blot images and additional binding curves for GATA1 mutants binding to 51_HAP sequence. **A.** Western blot images of nHU titrations with the 51_HAP sequence on cell extracts prepared of HA-GATA1 WT and mutant transfected HEK293T cells. **B.** Determined hyperbolic binding curves on titrations with the 51_HAP bait and with the mutant GATA1 proteins that are not shown in Figure 6. Error bars indicate standard deviation (SD) values.

Supplementary Figure S7. Additional images on the localization determination of different mutant versions of the GATA1. HEK293T cells were transfected with HA-GATA1 WT or mutant plasmids and immunostaining was carried out using an antibody recognizing the HA epitope (magenta), and the nucleus was visualized by using DAPI staining (green). Scale bar: 20 µm.

Supplementary Table 1. Biotinylated oligonucleotides used for native holdup. Each cases, the forward oligonucleotide was biotinylated at 5' end. All DNA oligonucleotides were produced by Eurofins Genomics (Eurofins Genomics Germany GmbH, Ebersberg, Germany) and were high-performance liquid chromatography (HPLC) purified. (5'-3' orientation, GATA1 recognition sequences and their haplotype versions are labelled green).

Supplementary Figure S1. Western blot images on transfected K562 cell extracts that were used in luciferase assay, to determine the HA-GATA1 expressions.

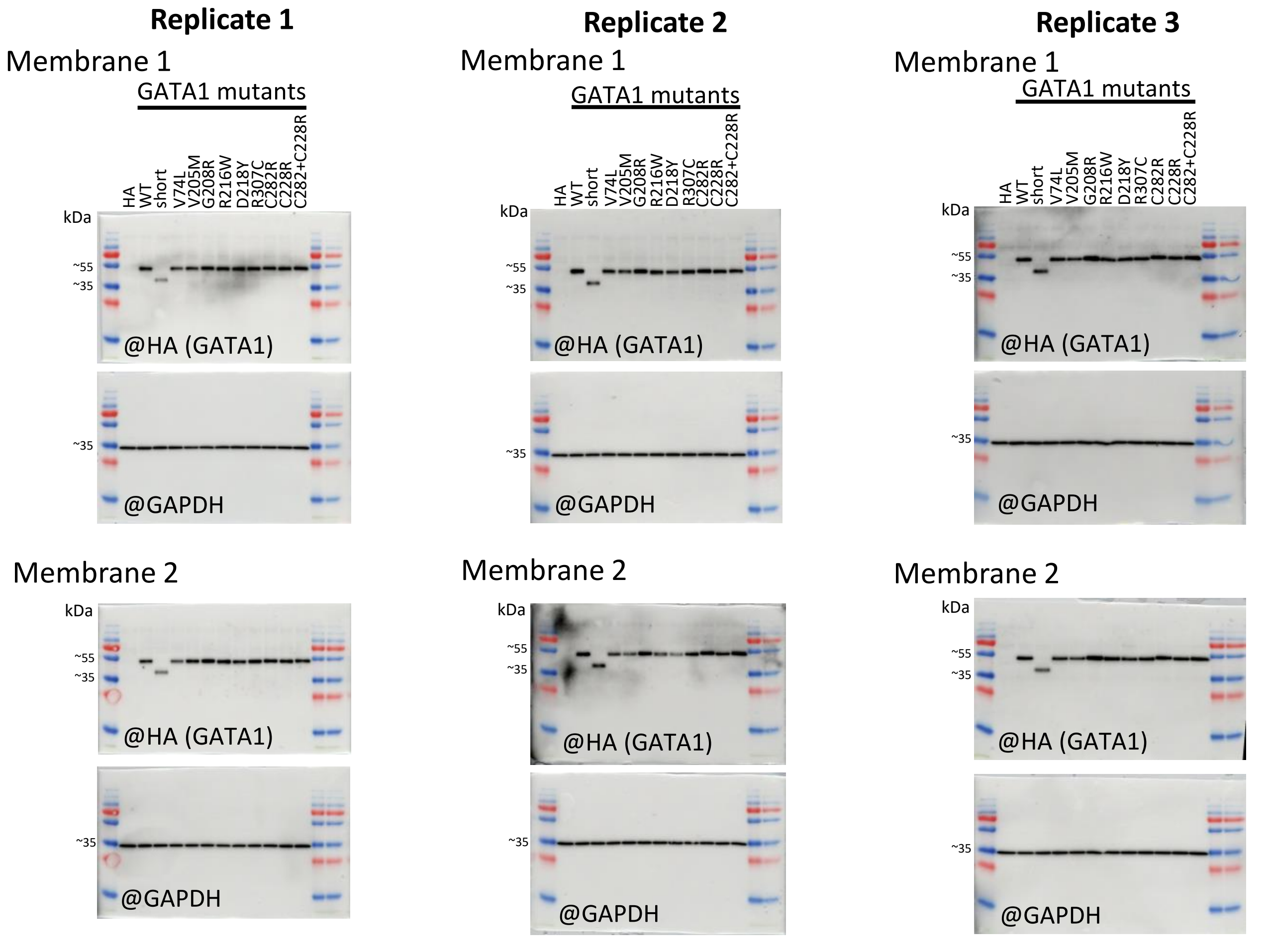

Supplementary Figure S2. Western blot images of single point nHU experiments with 51_WT, SCR1 and SCR2 sequences.

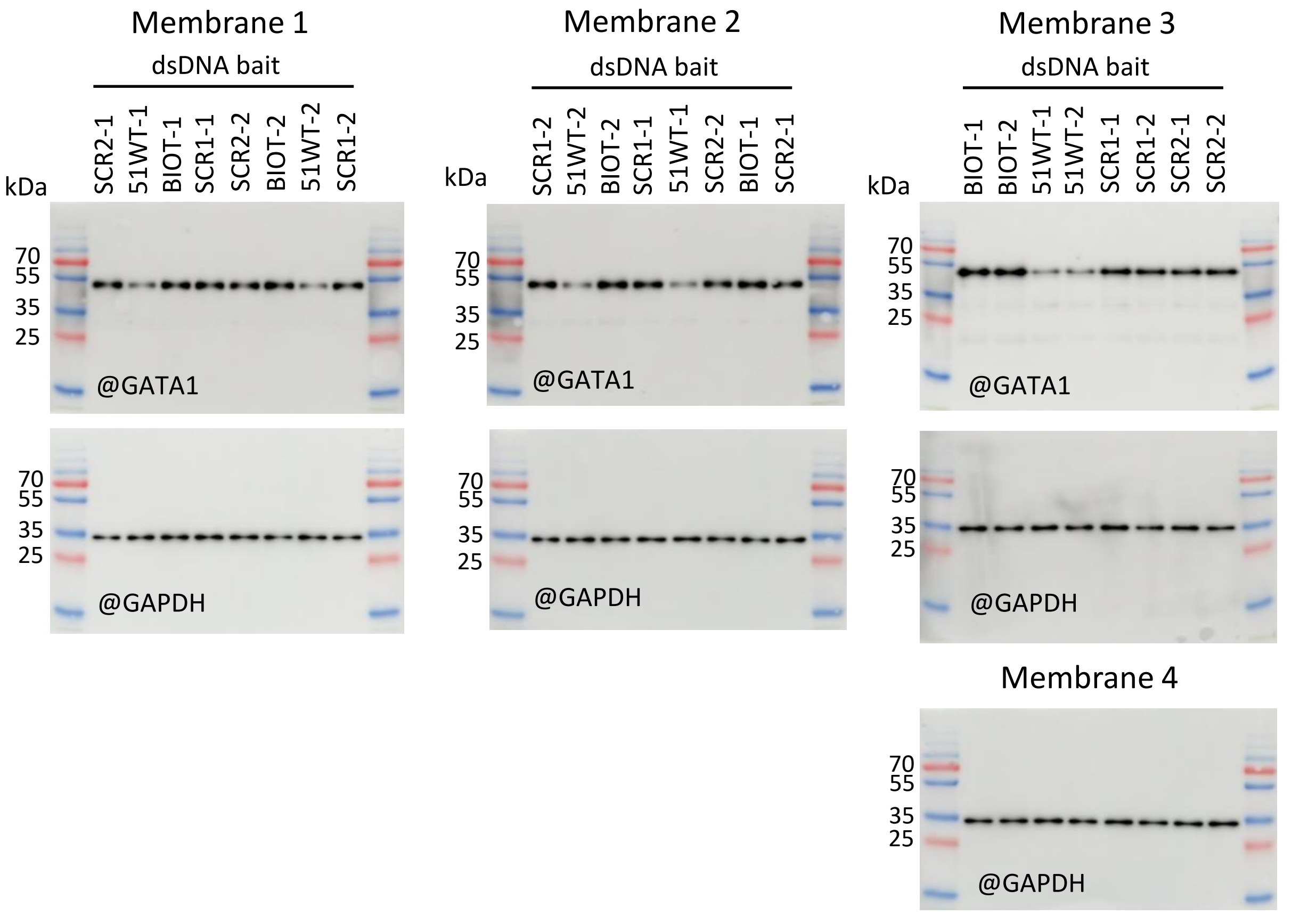

Supplementary Figure S3. Western blot images of titration nHU experiments with the 4945_WT, 4945_HAP, 51_WT, 51_HAP, or with the $CTRL_{PALINDROME}$ sequences on K562 cell extracts either at 1 mg/ml total protein concentration (upper panel) or 0.1 mg/ml total protein concentration (lower panel).

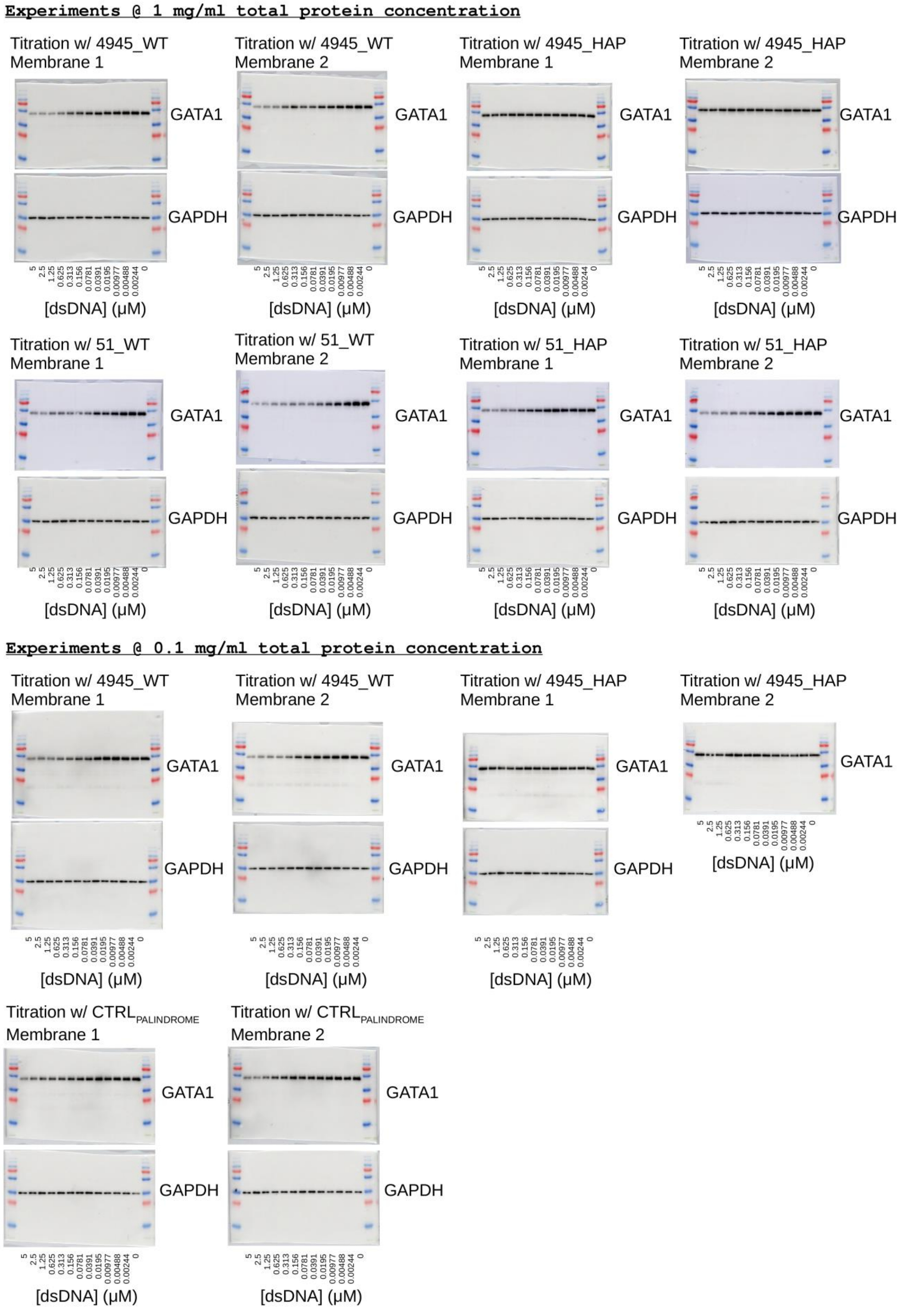

Supplementary Figure S4. Validation of transient expressions of the different mutant versions of GATA1 in HEK293T cells by Western blot. The HA antibody recognizes all mutants, but the GATA1 antibody doesn't recognize the short version of GATA1 (GATA1s) as the epitope recognized by the antibody might located at the N-terminal part of the protein.

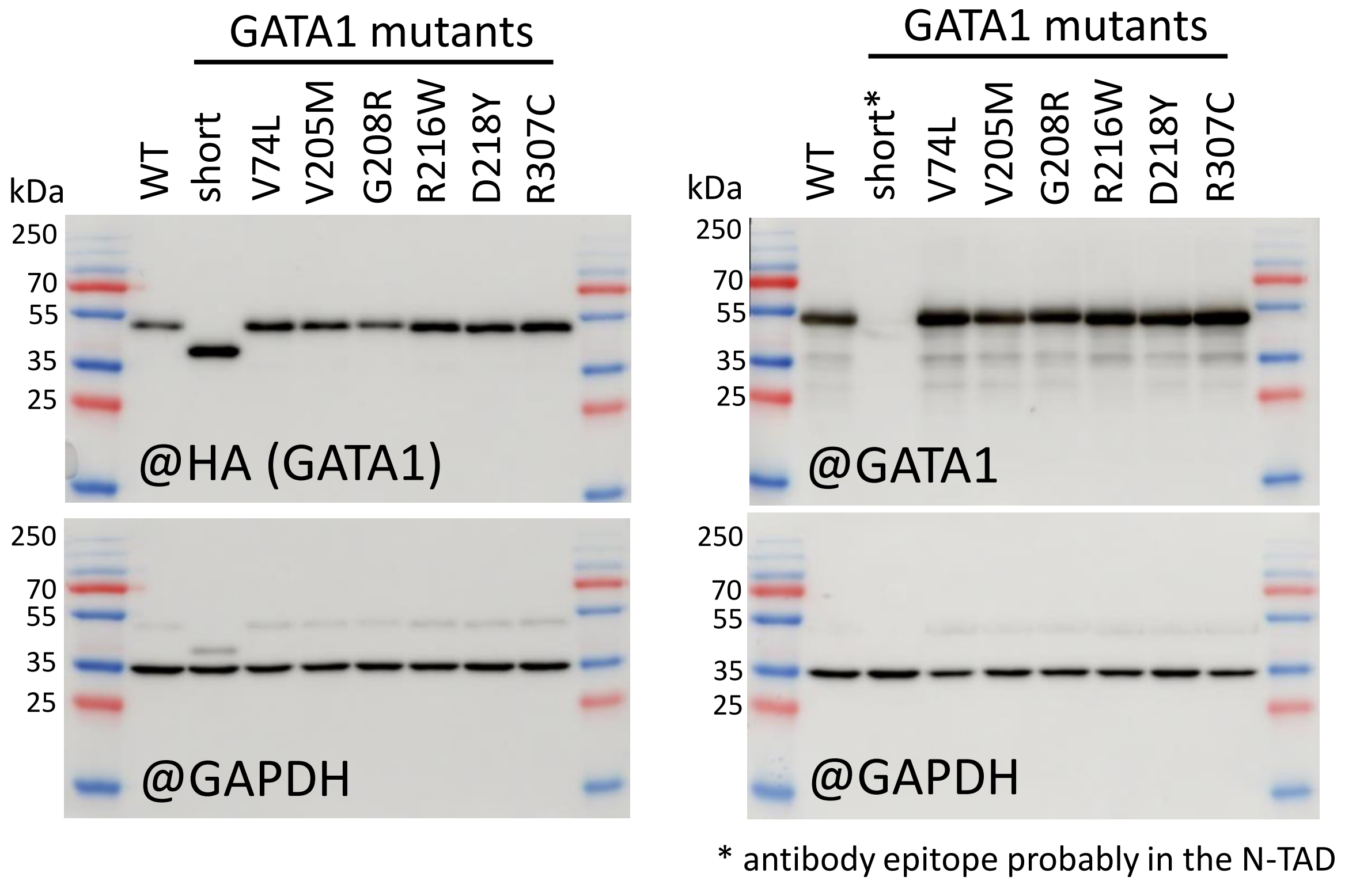

Supplementary Figure S5. Raw Western-blot images and additional binding curves for GATA1 mutants binding to 51_WT sequence. **A.** Western blot images of nHU titrations with the 51_WT sequence on cell extracts prepared of HA-GATA1 WT and mutant transfected HEK293T cells. **B.** Determined hyperbolic binding curves on titrations with the 51_WT bait and with the mutant GATA1 proteins that are not shown in Figure 6. Error bars indicate standard deviation (SD) values (n=2).

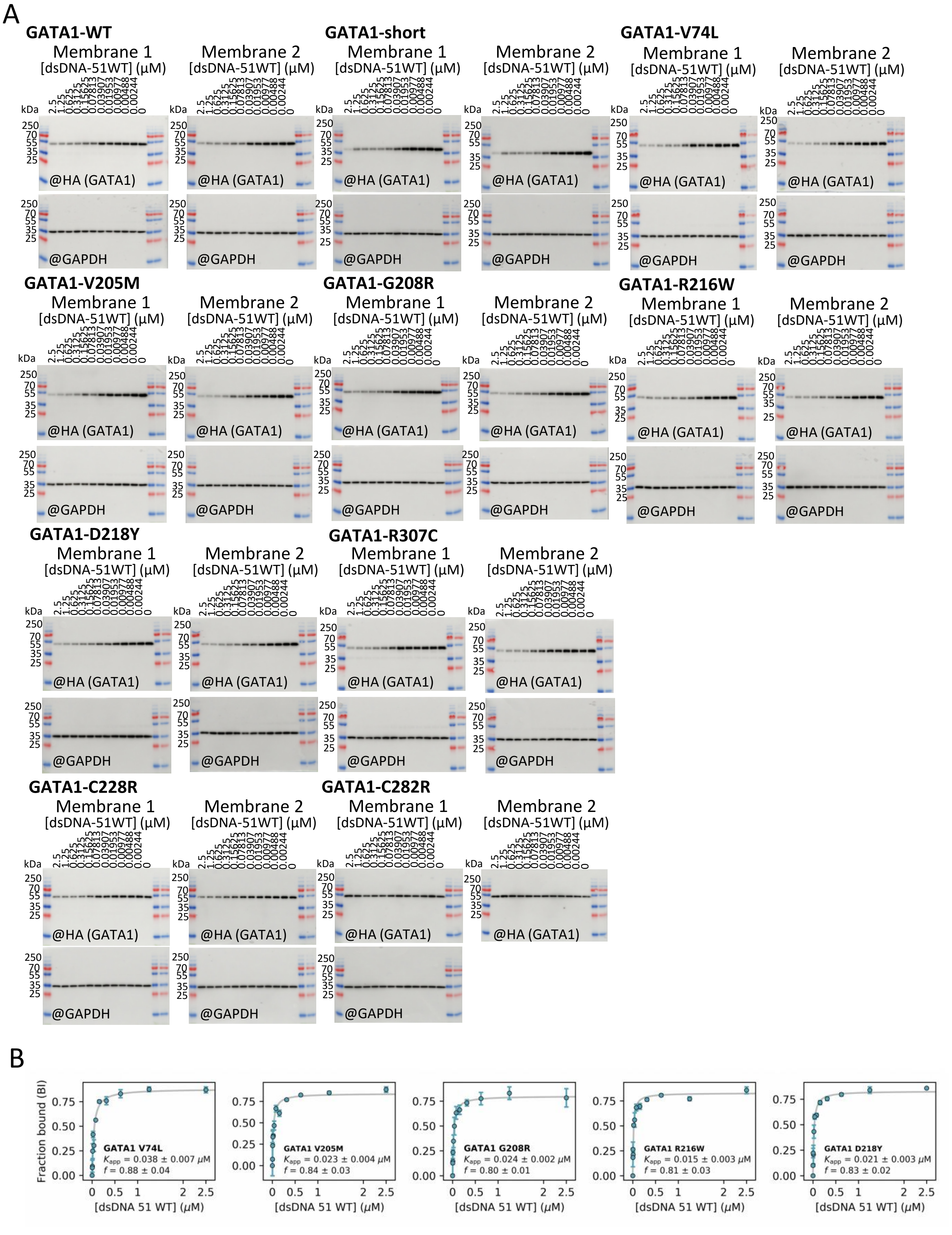

Supplementary Figure S6. Raw Western-blot images and additional binding curves for GATA1 mutants binding to 51_HAP sequence. **A.** Western blot images of nHU titrations with the 51_HAP sequence on cell extracts prepared of HA-GATA1 WT and mutant transfected HEK293T cells. **B.** Determined hyperbolic binding curves on titrations with the 51_HAP bait and with the mutant GATA1 proteins that are not shown in Figure 6. Error bars indicate standard deviation (SD) values (n=2).

A

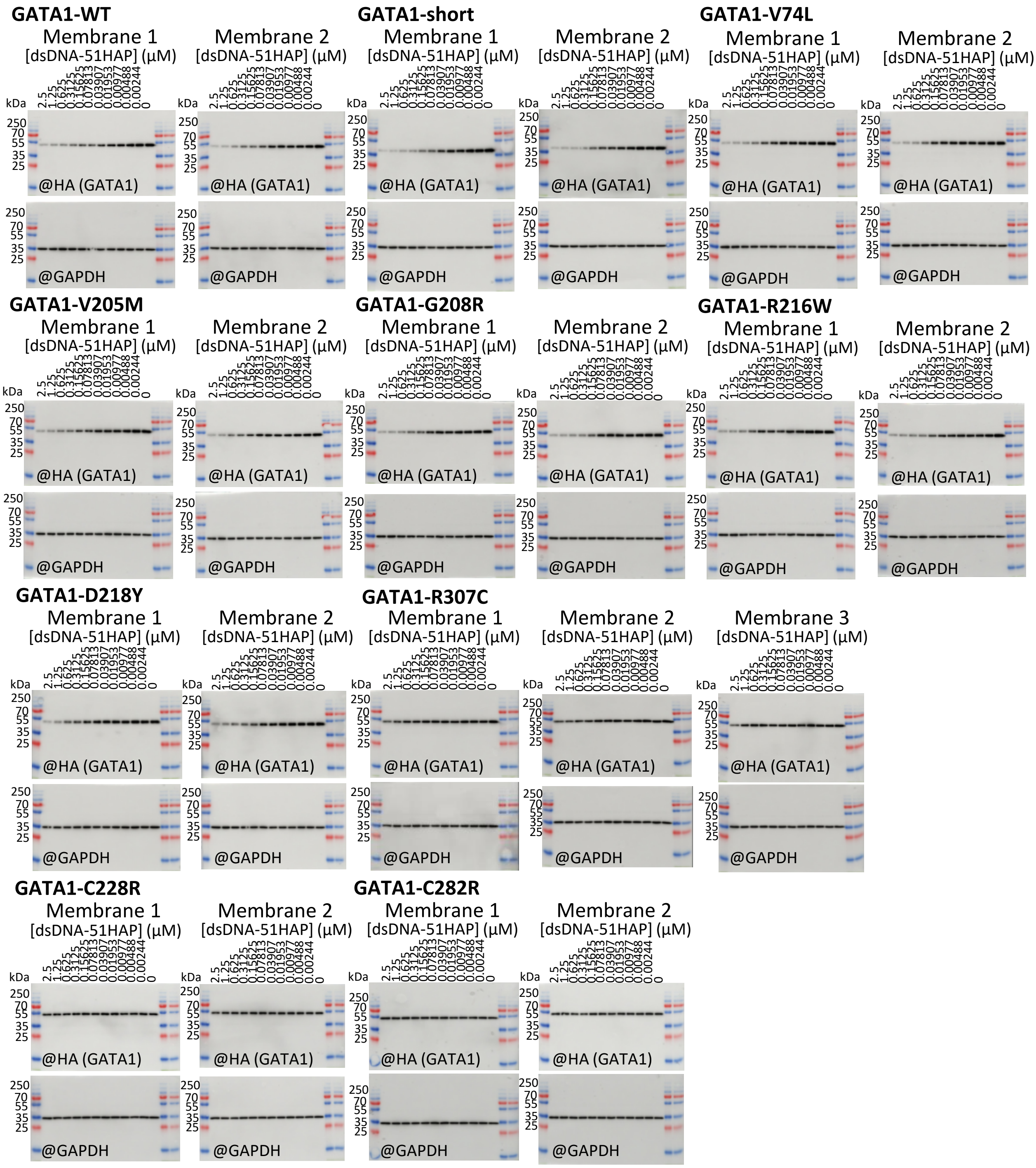

B

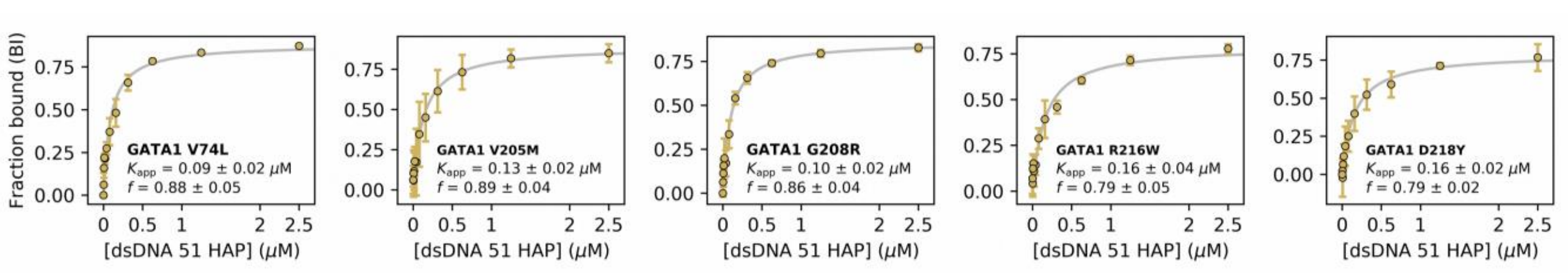

Supplementary Figure S7. Additional images on the localization determination of different mutant versions of the GATA1. HEK293T cells were transfected with HA-GATA1 WT or mutant plasmids and immunostaining was carried out using an antibody recognizing the HA epitope (magenta), and the nucleus was visualized by using DAPI staining (green). Scale bar: 20 µm.

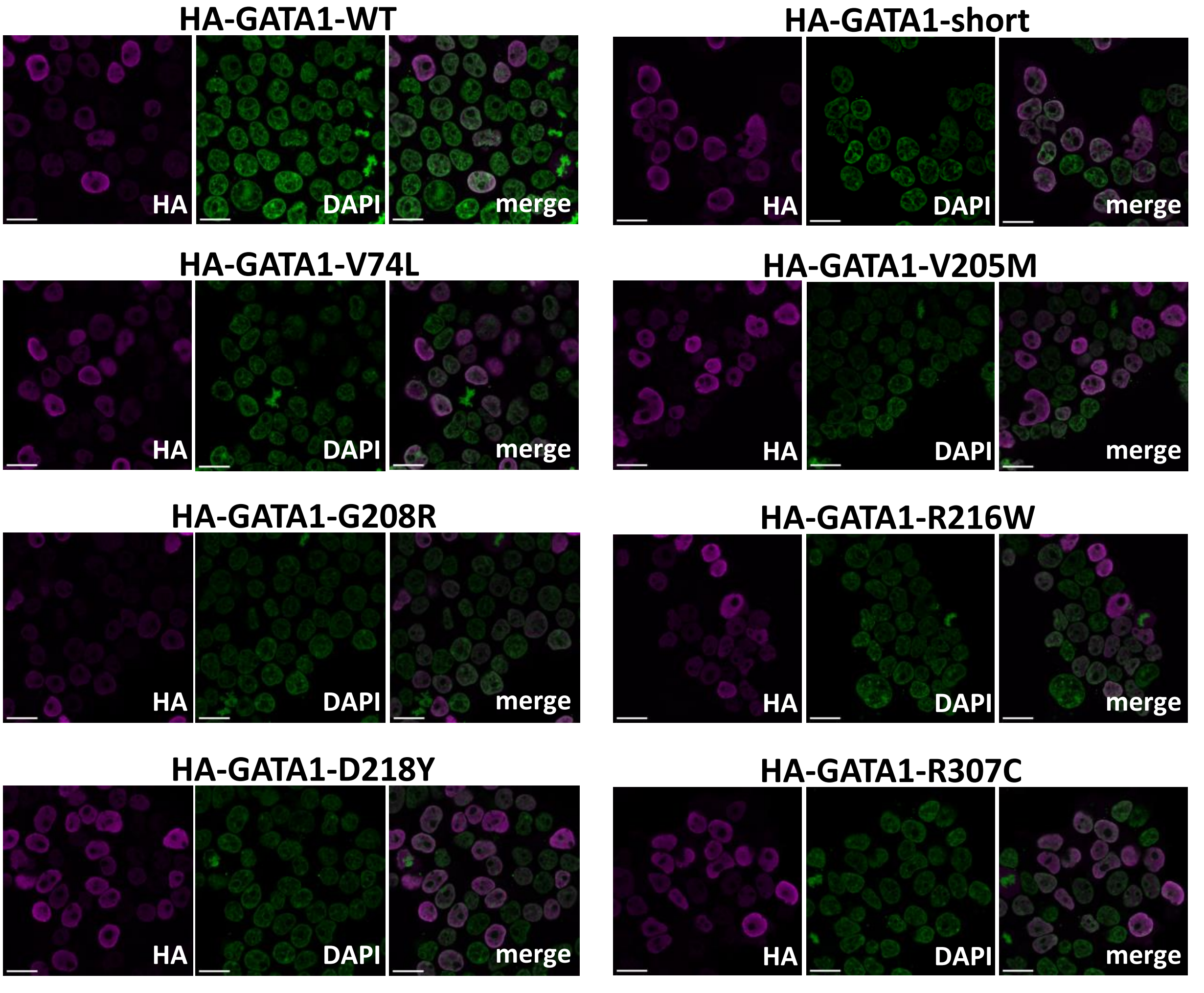

Supplementary Table 1. Biotinylated oligonucleotides used for native holdup. Each cases, the forward oligonucleotide was biotinylated at 5' end. All DNA oligonucleotides were produced by Eurofins Genomics (Eurofins Genomics Germany GmbH, Ebersberg, Germany) and were high-performance liquid chromatography (HPLC) purified. (5'-3' orientation, GATA1 recognition sequences and their haplotype versions are labelled green).

**Name**	**Forward (Biotinylated)**	**Reverse**
4945_WT	TCAGCCCTCCG**TATC**GTCACCTACAC	GTGTAGGTGAC**GATA**CGGAGGGCTGA
4945_HAP	TCAGCCCTCCG**TTTT**GTCACCTACAC	GTGTAGGTGAC**AAAA**CGGAGGGCTGA
51_WT	GAATTGAGAGG**TATC**T**TATC**GCTCCCACTCC	GGAGTGGGAGC**GATA**A**GATA**CCTCTCAATTC
51_HAP	GAATTGAGAGG**TATC**T**TACC**GCTCCCACTCC	GGAGTGGGAGC**GGTA**A**GATA**CCTCTCAATTC
SCR1	CCACTCTGTTCTAGAGAAGCCTTCGTGAACT	AGTTCACGAAGGCTTCTCTAGAACAGAGTGG
SCR2	ATGCCATTGCTTCATGCACGTATAGCCACTG	CAGTGGCTATACGTGCATGAAGCAATGGCAT
$CTRL_{palindrome}$	GCGC**TATC**A**GATA**AGGCCTTG	CAAGGCCT**TATC**T**GATA**GCGC
